# Identification of mitophagy-related genes with diagnostic value in focal segmental glomerulosclerosis using bioinformatics analysis

**DOI:** 10.1016/j.isci.2026.118015

**Published:** 2026-10-23

**Authors:** Jianan Ma, Tianshuai Ma, Yongrui Zhang, Haijian Wei

**Affiliations:** 1 Department of Pathology, School of Basic Medical Sciences, Shandong Medical and Pharmaceutical University, Yantai, Shandong 264000, China; 2 Intensive Care Unit, The First Affiliated Hospital of Harbin Medical University, Harbin, Heilongjiang 150000, China; 3 Department of Urology, The First Hospital of Jilin University, Changchun, Jilin 130000, China; 4 Department of Organ Transplantation, The Affiliated Yantai Yuhuangding Hospital of Qingdao University, Yantai, Shandong 264000, China

**Keywords:** focal segmental glomerulosclerosis, mitophagy, podocyte injury, bioinformatics, immune infiltration

## Abstract

Focal segmental glomerulosclerosis (FSGS), a major cause of end-stage renal disease, involves podocyte injury and immune dysregulation. We integrated GEO transcriptomic datasets to identify 839 differentially expressed genes and 33 differential mitophagy-related genes via differential expression analysis and weighted gene co-expression network analysis. Machine learning identified six candidate diagnostic biomarkers, SLIRP, BLVRB, ATP6V1F, COX5B, TMEM14A, and TRMT112, with moderate-to-good discriminatory capacity across cohorts. xCell analysis revealed extensive renal immune remodeling in FSGS with expanded pro-inflammatory cells, diminished naive T cells and Tregs, and tight correlations between hub genes and immune profiles. Single-cell RNA-sequencing analysis suggested a trend of downregulation of hub genes in FSGS podocytes, alongside suppressed transcriptomic signatures of mitochondrial energy metabolism and mitophagy-related pathways. qRT-PCR and western blot validated their downregulation at the whole-renal-tissue level in FSGS mouse models. These six genes may serve as diagnostic candidates and provide research clues for exploring mitophagy mechanisms in FSGS.

## Highlights

• Integrative analysis identified six mitophagy-related genes as candidate FSGS biomarkers
• The six-gene signature correlated with renal immune dysregulation in FSGS
• Single-cell analysis and mouse models revealed downregulation of the six genes in FSGS
• The six-gene signature showed moderate-to-good diagnostic performance across cohorts

## Graphical abstract

**Figure:**
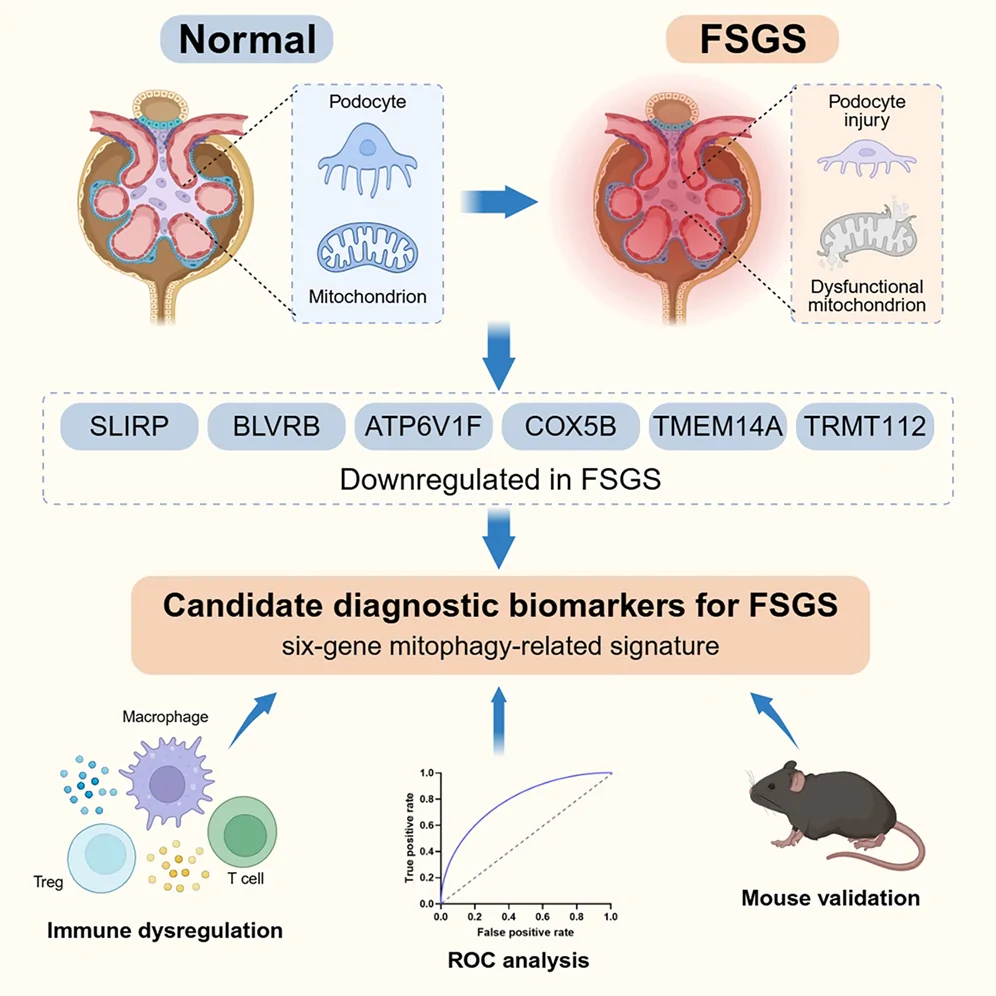

## Teaser

Clinical genetics; Omics

## Introduction

Focal segmental glomerulosclerosis (FSGS) is a common clinicopathological syndrome characterized by focal and segmental glomerulosclerosis, presenting primarily with proteinuria or nephrotic syndrome.^1^ Globally, FSGS is a leading cause of end-stage renal disease (ESRD).^2^^,^^3^^,^^4^ Current treatments, including corticosteroids, immunosuppressive therapies, and kidney transplantation, often yield unsatisfactory outcomes due to immune alterations in proinflammatory or profibrotic environments, with FSGS recurring in some cases post-transplantation.^4^ FSGS affects diverse populations, with incidence rates of 7–10% in children and 20–30% in adults with nephrotic syndrome.^5^ The primary initiating lesion in FSGS occurs in podocytes, highly differentiated epithelial cells critical to the glomerular filtration barrier.^6^ Podocytes have limited regenerative capacity and are vulnerable to genetic defects, immune factors, infections, metabolic stress, and hemodynamic injury, leading to irreversible damage manifested as podocyte loss, glomerular basement membrane exposure, and capillary collapse.^1^^,^^6^ Data suggest that podocyte depletion exceeding 20% mediates FSGS development, with depletion beyond 40% triggering progressive glomerulosclerosis.^7^

Podocytes, with their complex structure and multifaceted functions, rely on substantial energy from glycolysis and mitochondrial metabolism to maintain functionality.^8^^,^^9^^,^^10^ Beyond energy provision, mitochondria regulate reactive oxygen species (ROS), intracellular calcium homeostasis, cell proliferation, and various forms of cell death, including apoptosis, necrosis, pyroptosis, and ferroptosis.^11^^,^^12^^,^^13^^,^^14^^,^^15^ Under stress, damaged or dysfunctional mitochondria generate excessive ROS and pro-apoptotic factors, necessitating selective degradation via mitophagy to maintain cellular homeostasis.^16^ Studies disrupting autophagy genes (e.g., ATG5 or ATG7) in mouse renal epithelial cells demonstrate that impaired autophagy leads to mild podocyte and tubular dysfunction within two months, FSGS-like pathology by four months, and renal failure by six months.^17^ In human idiopathic FSGS kidney biopsies, dysfunctional mitochondria with morphological changes, such as increased diameter, shortened length, and focal cristae loss, are prevalent, underscoring mitophagy’s role in mitochondrial quality control and cell survival.^17^ Thus, modulating mitochondrial quality control and mitophagy holds significant potential for ameliorating FSGS and delaying its progression to ESRD.

This study identifies differentially expressed genes (DEGs) between FSGS and control samples using two GEO datasets, employs weighted gene co-expression network analysis (WGCNA) to select FSGS-related module genes, and intersects these with mitophagy-related genes to obtain differentially expressed mitophagy-related genes (DMRGs). Through Gene Ontology (GO) and Kyoto Encyclopedia of Genes and Genomes (KEGG) enrichment analyses, we explored their biological functions and pathways. Machine learning (random forest and support vector machine) further screened six candidate genes, whose diagnostic performance was validated by internal and external datasets as well as a nomogram model. xCell immune infiltration analysis was used to characterize renal immune cell remodeling and the correlation between hub genes and immune profiles. Single-cell RNA-sequencing (scRNA-seq) data further inferred potential podocyte-related expression patterns and alterations in mitochondrial metabolic and mitophagy pathways. Finally, qRT-PCR together with western blot validated candidate gene expression trends using renal tissues from an *in vivo* FSGS mouse model, aiming to provide potential biomarkers for FSGS prevention and diagnosis.

## Results

### Identification of DEGs

Batch effects between GSE125779 and GSE129973 (Table 1) were normalized using the “sva” package, with PCA confirming effect removal (Figures 1A and 1B). The merged dataset included 28 control and 28 FSGS samples. Differential analysis identified 839 DEGs (|logFC| > 0.5, *P*-adjusted <0.05), with 276 upregulated and 563 downregulated genes, visualized in volcano plots (Figure 1C) and heatmaps (Figure 1D).

**Table 1:** The information of FSGS-related datasets from the GEO

GEO accession_[year]_	Platform	Species	Tissues	Control	FSGS	Group
GEO: GSE125779_[__2019__]_	GPL17586	Homo sapiens	kidney biopsy	8	8	merged dataset
GEO: GSE129973_[__2019__]_	GPL17586	Homo sapiens	kidney biopsy	20	20	merged dataset
GEO: GSE108109_[__2018__]_	GPL19983	Homo sapiens	kidney biopsy	6	30	validation dataset
GEO: GSE108112_[__2018__]_	GPL19983	Homo sapiens	kidney biopsy	5	46	validation dataset
GEO: GSE200818_[__2022__]_	GPL19983	Homo sapiens	kidney biopsy	5	54	validation dataset
GEO: GSE200828_[__2022__]_	GPL19983	Homo sapiens	kidney biopsy	6	31	validation dataset
GEO: GSE104948_[__2018__]_	GPL22945	Homo sapiens	kidney biopsy	18	16	validation dataset
GEO: GSE104954_[__2018__]_	GPL22945	Homo sapiens	kidney biopsy	21	17	validation dataset
GEO: GSE146912_[__2020__]_	GPL21103	Mus musculus	kidney tissue	3	2	scRNA-seq dataset

**Figure 1:**
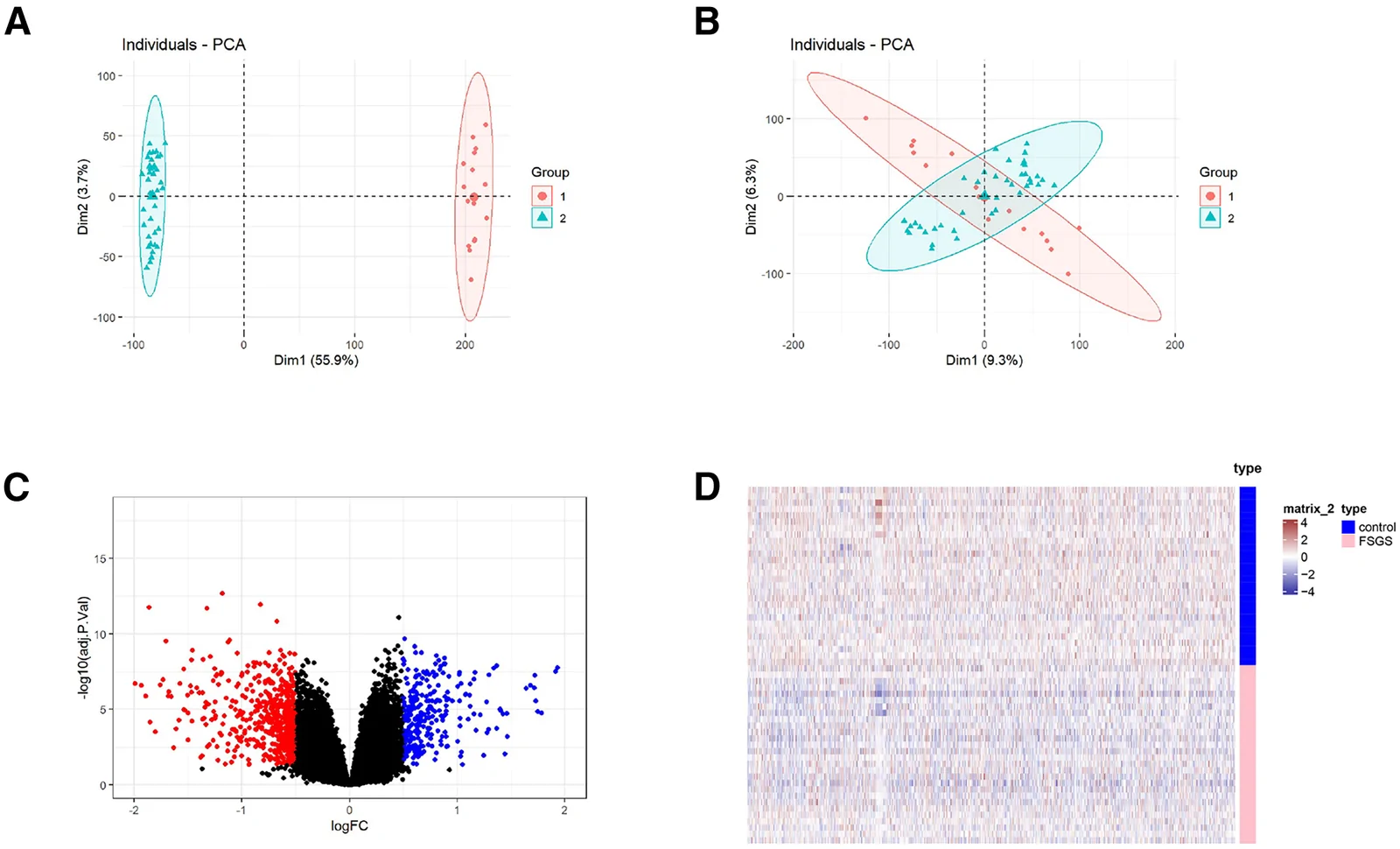
Identification of DEGs of the FSGS and control groups (A) PCA plot of the dataset (GSE125779 and GSE129973) before batch effect removal. (B) PCA plot after batch effect normalization. (C) Volcano plot of all DEGs in the merged dataset (GSE125779 and GSE129973). Blue dots indicate upregulated genes, red dots represent downregulated genes, and black dots denote genes with no significant change in expression. (D) Heatmap of expression matrix, contrasting control (blue) and FSGS (pink) samples.

### WGCNA and DMRGs screening

We conducted WGCNA using the top 25% genes obtained by variance analysis from the gene expression matrix containing 30,906 genes. Clustering removed outlier sample GSM3728803 (Figure 2A). A soft threshold of *β* = 9 achieved scale-free topology (*R*^2^ > 0.9) with high connectivity (Figure 2B), identifying 16 co-expression modules (Figure 2C). The blue module showed the strongest FSGS correlation (cor = −0.7522, *p* = 3.575e−11; Figure 2D), with high MM and GS correlation (cor = 0.74, *P* = 1e−192; Figure 2E). This module, containing 212 genes, was selected as the candidate module. Intersecting 839 DEGs, 212 module genes, and 5,150 mitophagy-related genes from GeneCards yielded 33 DMRGs (Figure 2F).

**Figure 2:**
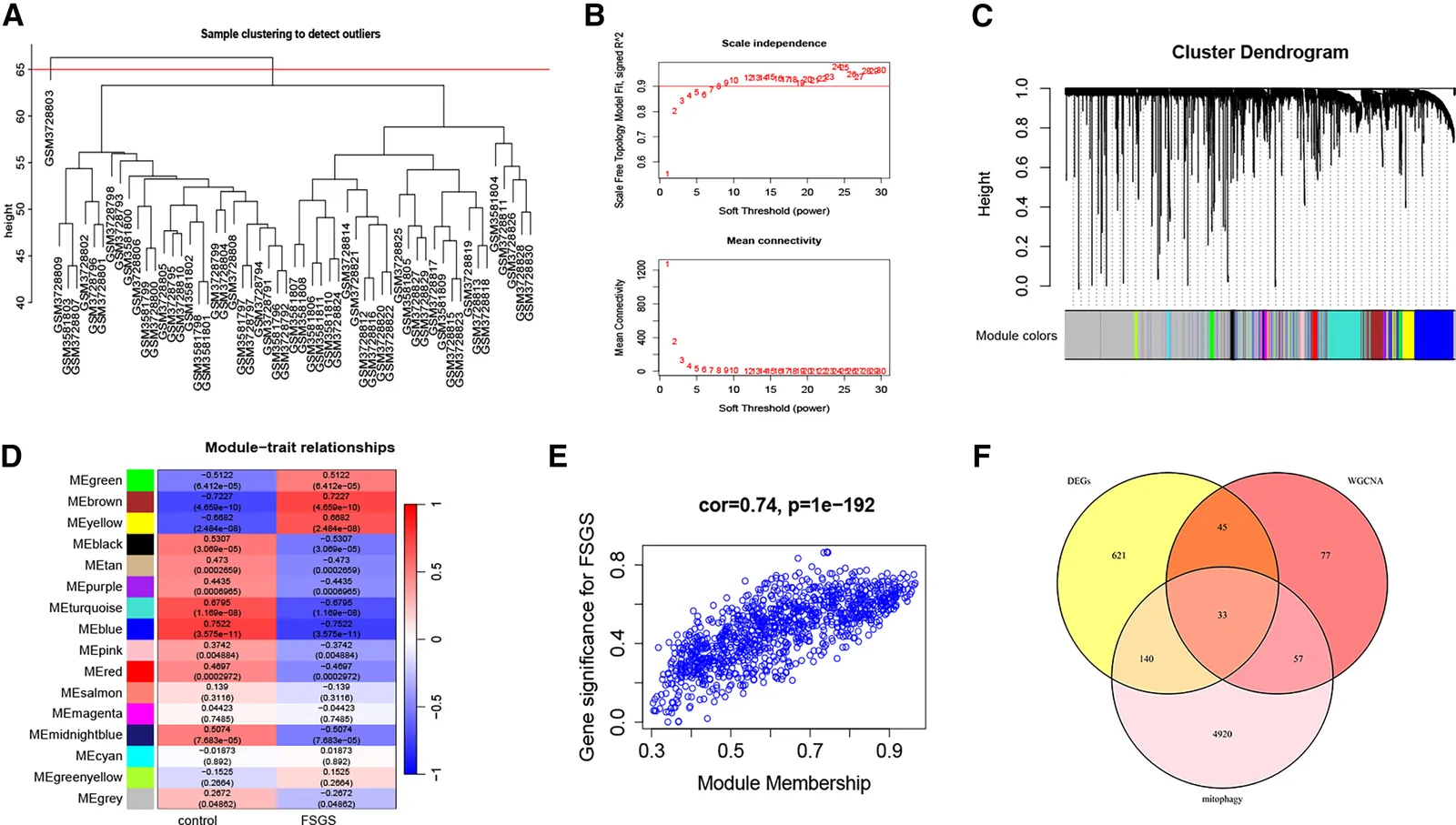
WGCNA and DMRGs screening (A) Clustering analysis of the samples. (B) Measurement of the soft threshold power based on the scale-free fit index (top) and mean connectivity (bottom). (C) Gene dendrogram with corresponding module colors identified by WGCNA. (D) Heatmap showing the module-trait relationships. (E) The scatterplot showing the correlation between FSGS and the blue module. (F) The Venn diagram representing the intersection of DEGs, WGCNA module genes, and the mitophagy-related genes.

### Functional enrichment analysis for DMRGs

GO analysis revealed DMRGs involvement in biological processes such as translation, hydrogen ion transmembrane transport, cytoplasmic translation, mitochondrial electron transport, and cytochrome *c* to oxygen (Figure 3A); cellular components including mitochondrion, mitochondrial inner membrane, ribonucleoprotein complex, ribosome, and cytosolic ribosome (Figure 3B); and molecular functions such as protein binding, RNA binding, structural constituent of ribosome, cytochrome *c* oxidase activity, and ribosome binding (Figure 3C). KEGG analysis linked DMRGs to pathways such as Parkinson’s disease, oxidative phosphorylation, Huntington’s disease, thermogenesis, and prion disease (Figure 3D).

**Figure 3:**
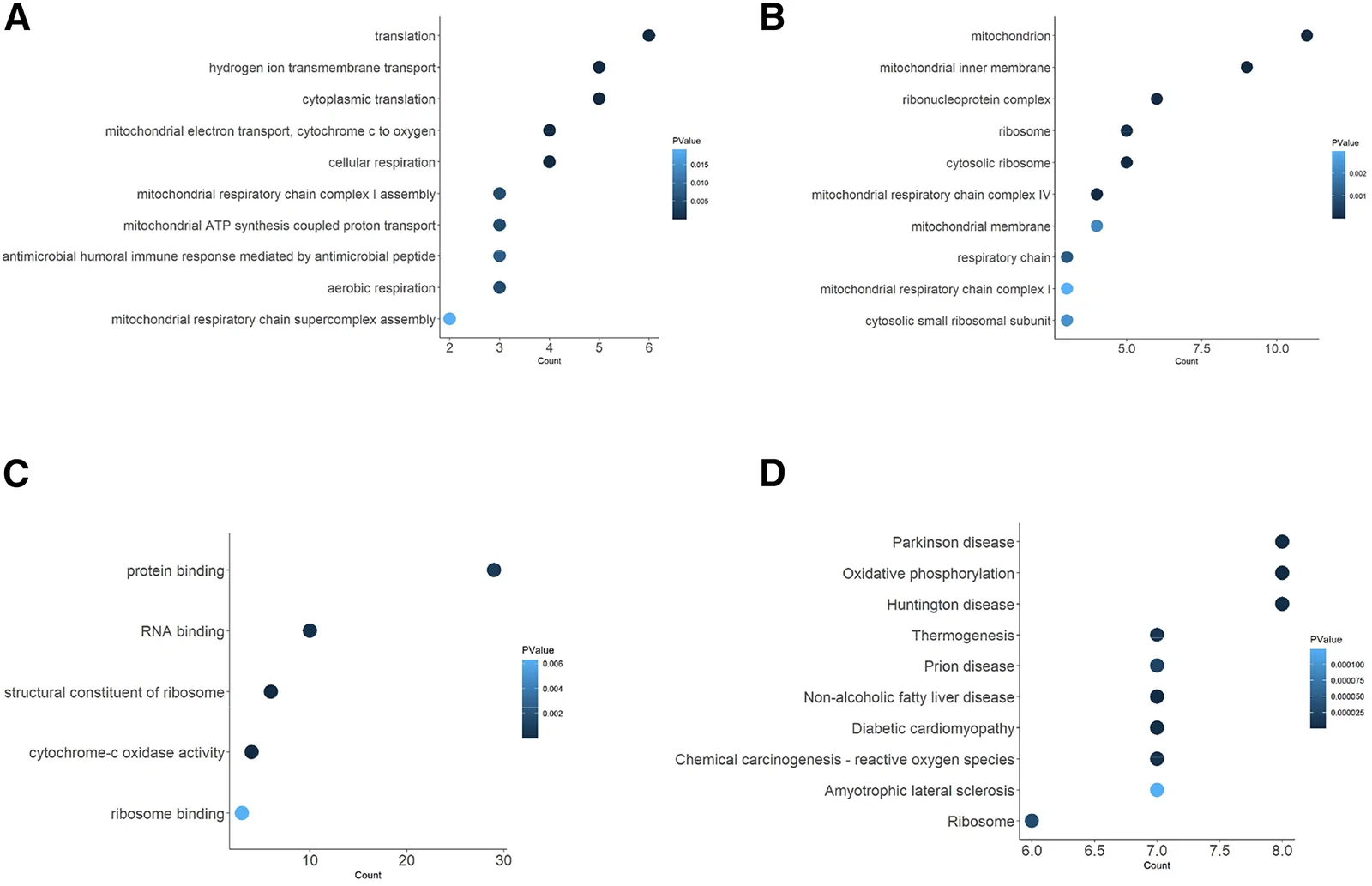
Function enrichment analysis of DMRGs Dot plot of GO enrichment analysis for (A) BP, (B) CC, (C) MF, and (D) KEGG pathway enrichment analysis, highlighting the most significant pathways.

### Identification of candidate genes

The 33 DMRGs were further screened using RF-RFE and SVM-RFE methods. Initially, the RF algorithm was applied (Figure 4A), ranking genes based on their importance (Figure 4B). Subsequently, RFE identified 13 key genes (Figure 4C). Similarly, the SVM method selected 14 key genes (Figure 4D). The intersection of these two models yielded six candidate genes most closely associated with the disease (Figure 4E): SRA Stem-Loop Interacting RNA Binding Protein (SLIRP), Biliverdin reductase B (BLVRB), ATPase H^+^ Transporting V1 Subunit F (ATP6V1F), Cytochrome *c* Oxidase Subunit 5B (COX5B), Transmembrane Protein 14A (TMEM14A), and tRNA Methyltransferase Activator Subunit 11–2 (TRMT112). These candidate genes are involved in various mitochondrial processes closely related to quality control and were subjected to further analysis.

**Figure 4:**
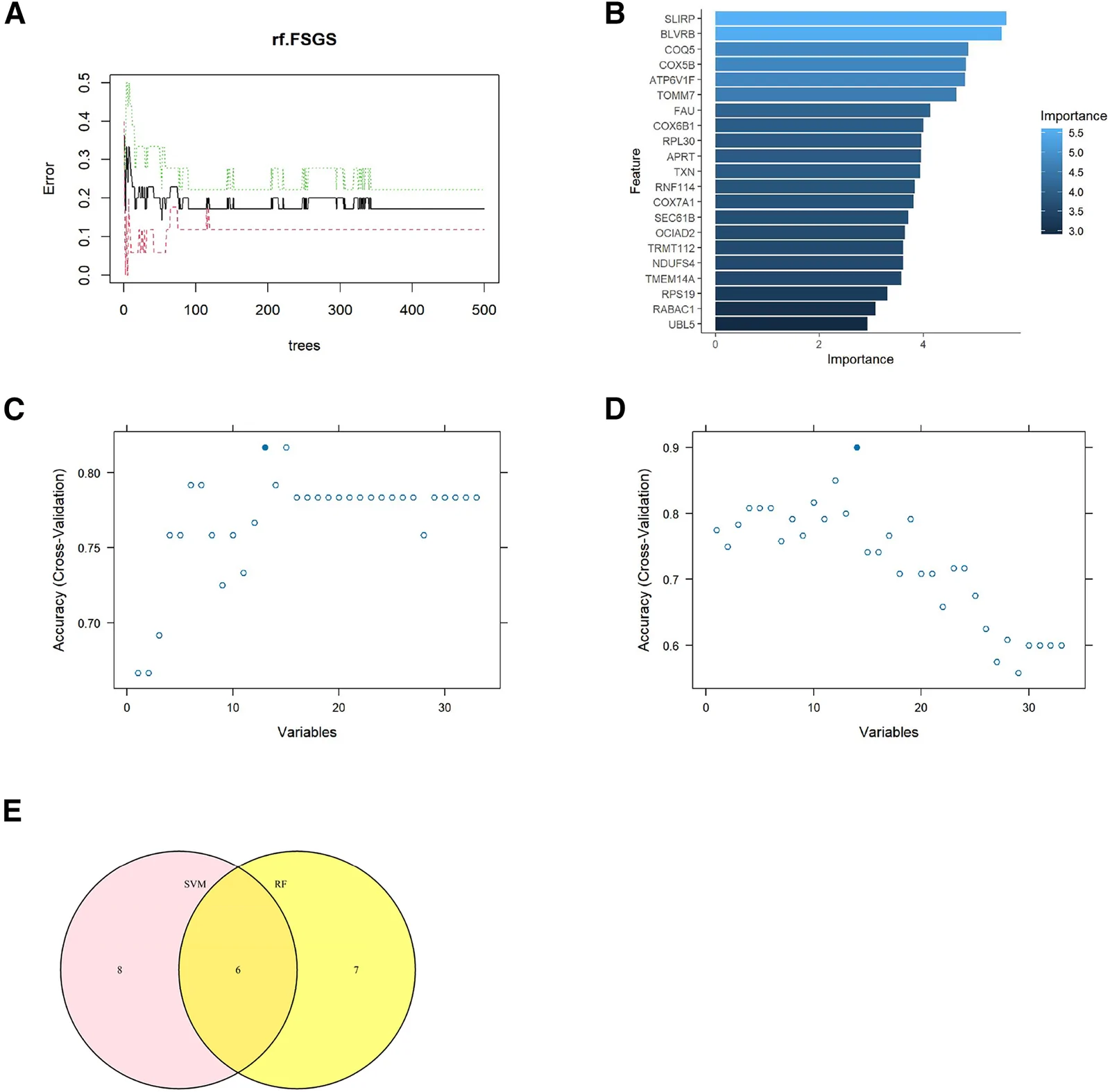
Identification of candidate genes (A) Random forest tree. (B) Gene importance ranking based on RF-RFE. (C) RF-RFE feature selection result plot. (D) SVM-RFE feature selection result plot. (E) Venn diagram of the intersection of the genes screened by RF-RFE and SVM-RFE.

To validate the biological validity of the six pre-identified core hub genes (ATP6V1F, BLVRB, COX5B, SLIRP, TMEM14A, TRMT112) in FSGS, we integrated six public FSGS kidney transcriptome datasets (total 239 samples: 50 healthy controls, 189 FSGS cases) for large-sample validation (Table 1). After batch correction with the ComBat algorithm, inter-dataset batch effects were effectively eliminated, and PCA showed clear separation between healthy control and FSGS samples (Figure S1A). 100 repetitions of 5-fold cross-validation showed the model had an average AUC of 0.932 (95% CI: 0.916–0.943), indicating stable discrimination capacity within this combined multi-cohort training set (Figure S1B). 1000 label permutation tests revealed the original model had an AUC of 0.942 (Figure S1C), which was significantly higher than the permutation null distribution (*p* < 0.001). This confirmed that the high classification performance was not caused by random effects or overfitting, and the results were statistically reliable. The intersection of core feature genes screened by RF-RFE and SVM-RFE fully included the six candidate genes of this study. Variable importance analysis showed these genes ranked high in both the mean Gini coefficient decrease of the RF model and the standardized variable weight of the SVM model. They were core feature genes for distinguishing FSGS from healthy controls, further verifying the biological significance and classification robustness of this gene set (Figure S1D).

### Establishment of the predictive model for FSGS

A nomogram model was constructed using the six candidate genes to predict FSGS risk (Figure 5A). Individual gene expression scores were summed to assess total FSGS risk, with high SLIRP and COX5B expression and low BLVRB, ATP6V1F, TRMT112, and TMEM14A expression indicating higher risk. This finding confirms the effectiveness of the model in predicting the occurrence of FSGS.

**Figure 5:**
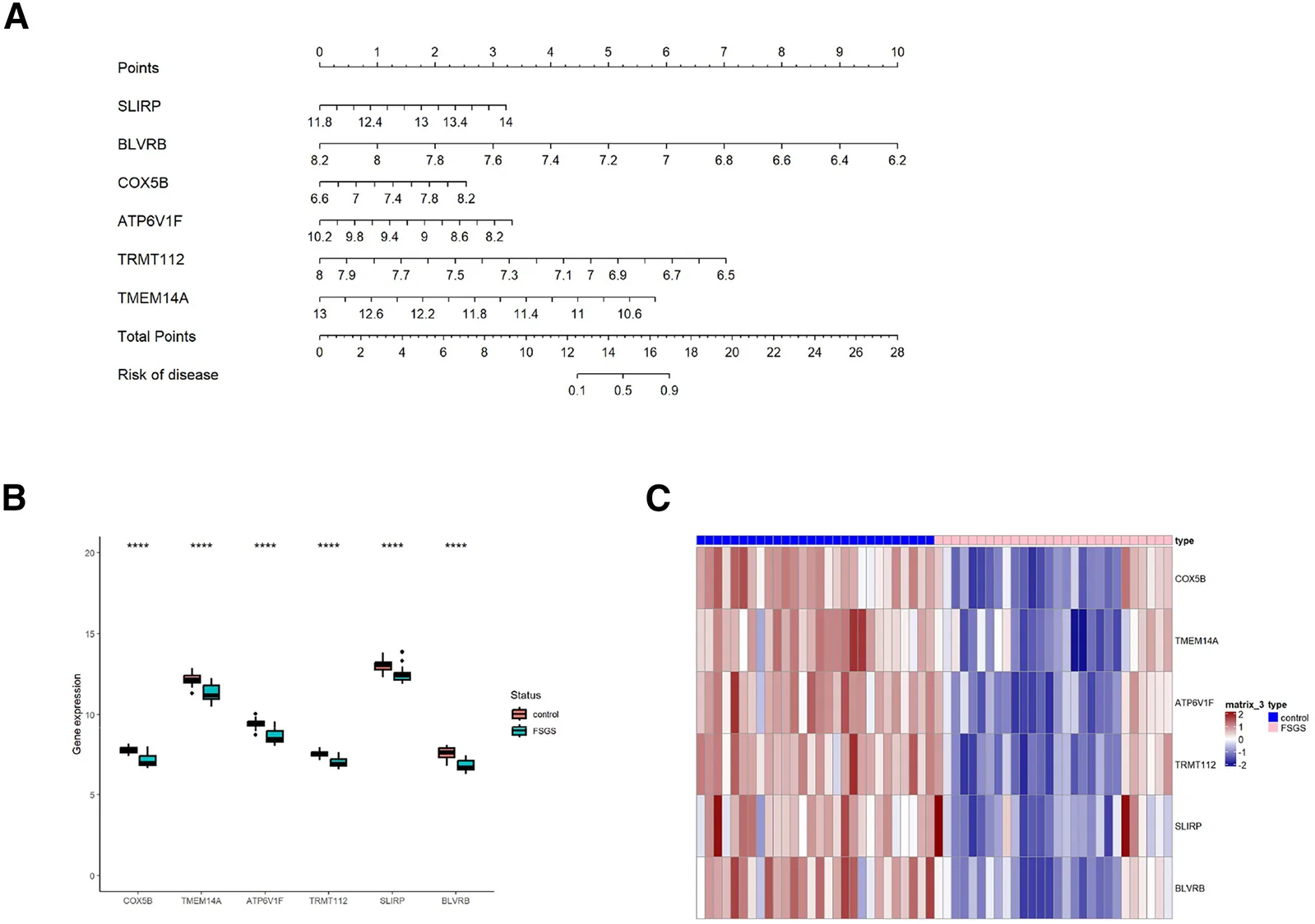
Construction of a predictive nomogram model and expression levels of candidate genes (A) Nomogram for assessing the risk of FSGS occurrence. (B) Boxplot and (C) Heatmap of the expression levels of six candidate genes in control and FSGS groups (merged dataset, *n* = 56; 28 control, 28 FSGS). Data were analyzed using the Wilcoxon rank-sum test. ∗*p* < 0.05, ∗∗*p* < 0.01, ∗∗∗*p* < 0.001, and ∗∗∗∗*p* < 0.0001.

Meanwhile, differential expression of six candidate genes was validated in the gene expression dataset, as shown in Figures 5B and 5C. Specifically, compared with the control group, the expression levels of the six candidate genes in the FSGS group were significantly downregulated (*p* < 0.05).

### Immune infiltration analysis

xCell immune infiltration deconvolution analysis was performed to evaluate differences in renal immune microenvironment between FSGS and control tissues. The results showed significant immune dysregulation in FSGS lesions. Specifically, FSGS samples exhibited markedly increased infiltration of pro-inflammatory and effector immune cells, including dendritic cells (DC), activated dendritic cells (aDC), CD4 effector memory T cells (CD4 Tem cells), CD8 effector memory T cells (CD8 Tem cells), eosinophils, macrophages, M2 macrophages, natural killer T cells (NKT cells), and gamma delta T cells (Tgd cells). In contrast, naive CD4 T cells, naive CD8 T cells, neutrophils, and regulatory T cells (Tregs) were significantly depleted in FSGS tissues (Figure 6A).

**Figure 6:**
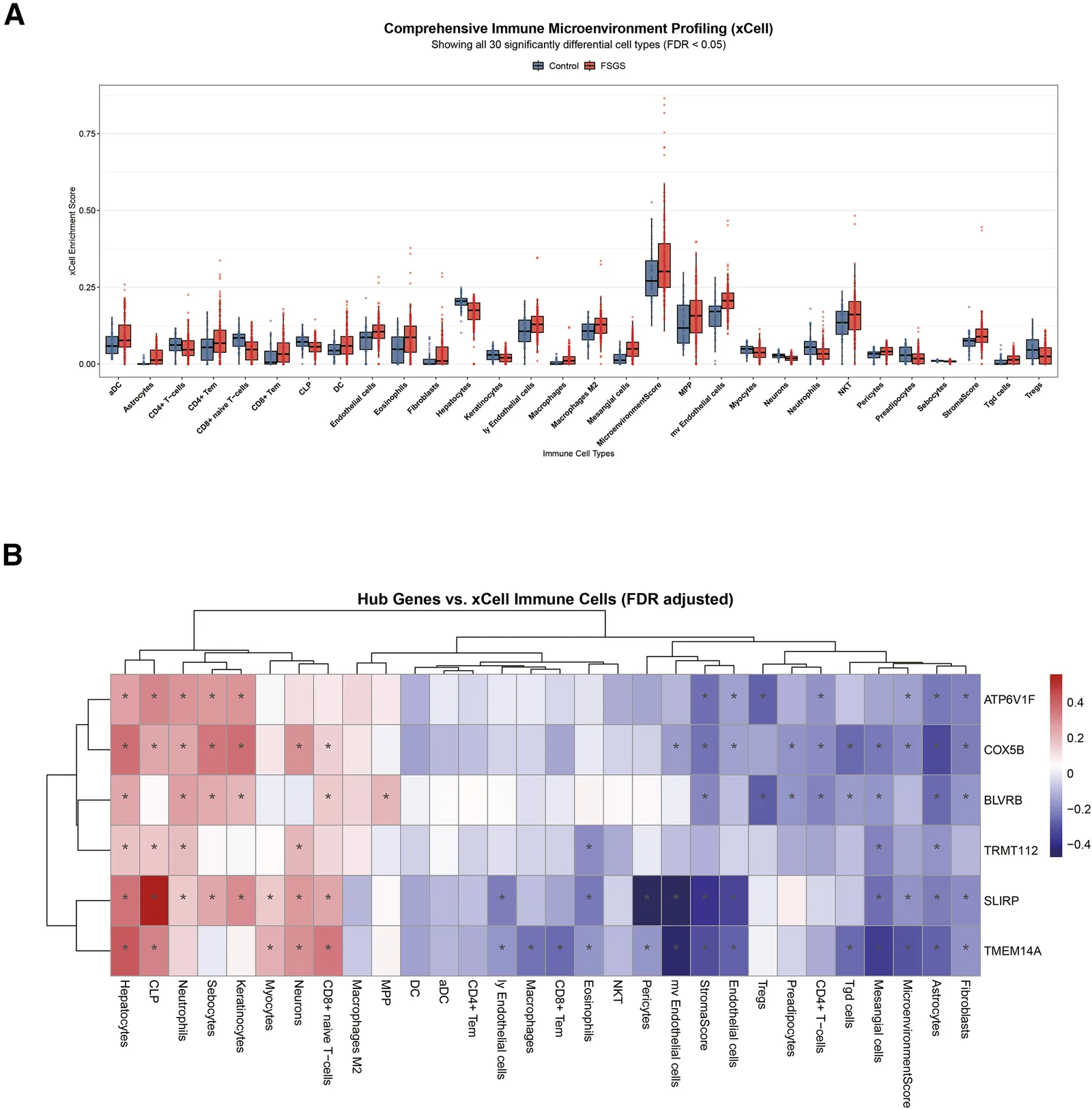
Analysis of Immune Cell Infiltration (A) Boxplot showing differentially enriched immune and stromal cell types between healthy control and FSGS groups, analyzed using the xCell algorithm (*n* = 239; 50 control, 189 FSGS). Red indicates cell types significantly enriched in FSGS; blue indicates those significantly depleted. (B) Heatmap shows Pearson’s correlation coefficients between the six core hub genes and differentially enriched cell types. Red indicates positive correlation, blue indicates negative correlation, with color intensity reflecting correlation strength. All statistical analyses were adjusted for multiple comparisons using the Benjamini-Hochberg method. Data were analyzed using the Wilcoxon rank-sum test (A) and Pearson’s correlation analysis (B). ∗*p* < 0.05.

Further correlation analysis of the six candidate hub genes with immune cell infiltration demonstrated significant associations with various immune cells in the renal microenvironment (Figure 6B). On the positive correlation side, ATP6V1F, COX5B, BLVRB, TRMT112, and SLIRP were positively correlated with neutrophils, while COX5B, BLVRB, SLIRP, and TMEM14A correlated positively with naive CD8 T cells. In terms of negative correlations, TMEM14A showed inverse associations with macrophages and CD8 Tem cells. TRMT112, SLIRP, and TMEM14A were negatively correlated with eosinophils; ATP6V1F and BLVRB correlated inversely with Tregs. Additionally, ATP6V1F, COX5B, and BLVRB were negatively associated with naive CD4 T cells, whereas COX5B, BLVRB, and TMEM14A correlated negatively with Tgd cells.

### Validation of candidate genes by ROC curve

We evaluated the specificity and sensitivity of six candidate genes in predicting FSGS occurrence by generating ROC curves using an internal dataset (merged GSE125779 and GSE129973 expression data) and external datasets (GSE104948 and GSE104954) (Table 1). The internal dataset validation results, shown in Figure 7A, revealed AUC values for SLIRP, BLVRB, ATP6V1F, COX5B, TMEM14A, and TRMT112 of 0.852, 0.936, 0.927, 0.902, 0.913, and 0.907, respectively. In the external dataset GSE104948 (Figure 7B), the AUC values for these genes were 0.774, 0.597, 0.618, 0.757, 0.642, and 0.878, respectively, while in GSE104954 (Figure 7C), the AUC values were 0.821, 0.739, 0.616, 0.706, 0.709, and 0.625. Overall, the six genes maintained acceptable predictive power across external cohorts, though moderate AUC values were observed for a small number of genes in isolated datasets.

**Figure 7:**
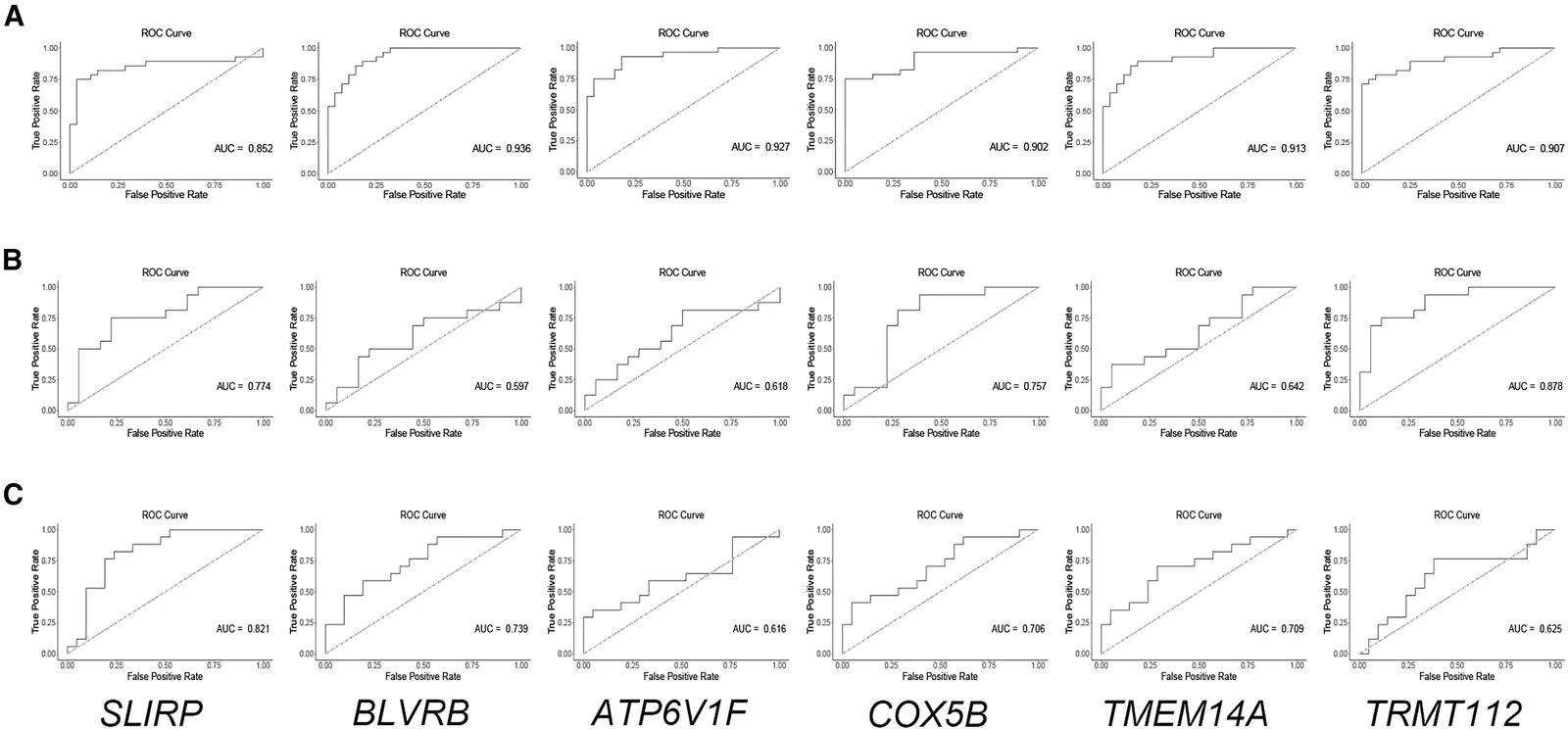
Validation of the diagnostic value of candidate genes ROC curves evaluating the diagnostic performance of SLIRP, BLVRB, COX5B, ATP6V1F, TMEM14A, and TRMT112 in (A) the internal dataset (merged expression matrix of GSE125779 and GSE129973), (B) the external dataset GSE104948, and (C) the external dataset GSE104954.

### Association between the six-gene signature and mitophagy-related transcriptomic alterations

To clarify the biological relevance of the six hub genes to disordered mitophagy in FSGS lesions, multi-level transcriptome mining was carried out based on the six public glomerular bulk RNA-seq cohorts mentioned above (Figure S2).

The pooled correlation heatmap revealed that most hub genes exhibited consistent positive correlations with canonical mitophagy molecules (PINK1, MAP1LC3B, SQSTM1, TOMM20, COX4I1, BNIP3, BNIP3L) (Figure S2A). GSVA results based on two independent mitophagy gene sets further validated that the activity of the mitophagy pathway was positively correlated with the six-gene signature score in all cohorts (Figure S2B). Consistent with the above findings, pre-ranked GSEA using co-expressed genes of the signature showed obvious enrichment in the autophagy-lysosome pathway, mitochondrial morphological organization, mitochondrial membrane potential regulation, and reactive oxygen species metabolic processes (Figure S2C).

Collectively, this multi-angle transcriptional evidence demonstrated that the six-gene signature was closely correlated with transcriptomic alterations of mitophagy and mitochondrial function pathways in human FSGS glomeruli.

### Single-cell transcriptomic analysis of podocyte pathology in adriamycin-induced FSGS mice

To explore potential podocyte-associated transcriptomic signatures and core injury mechanism under FSGS pathology at single-cell resolution, we performed scRNA-seq analysis on kidney tissues from adriamycin-induced FSGS model mice and normal control mice (Table 1), with the results presented in Figure 8.

**Figure 8:**
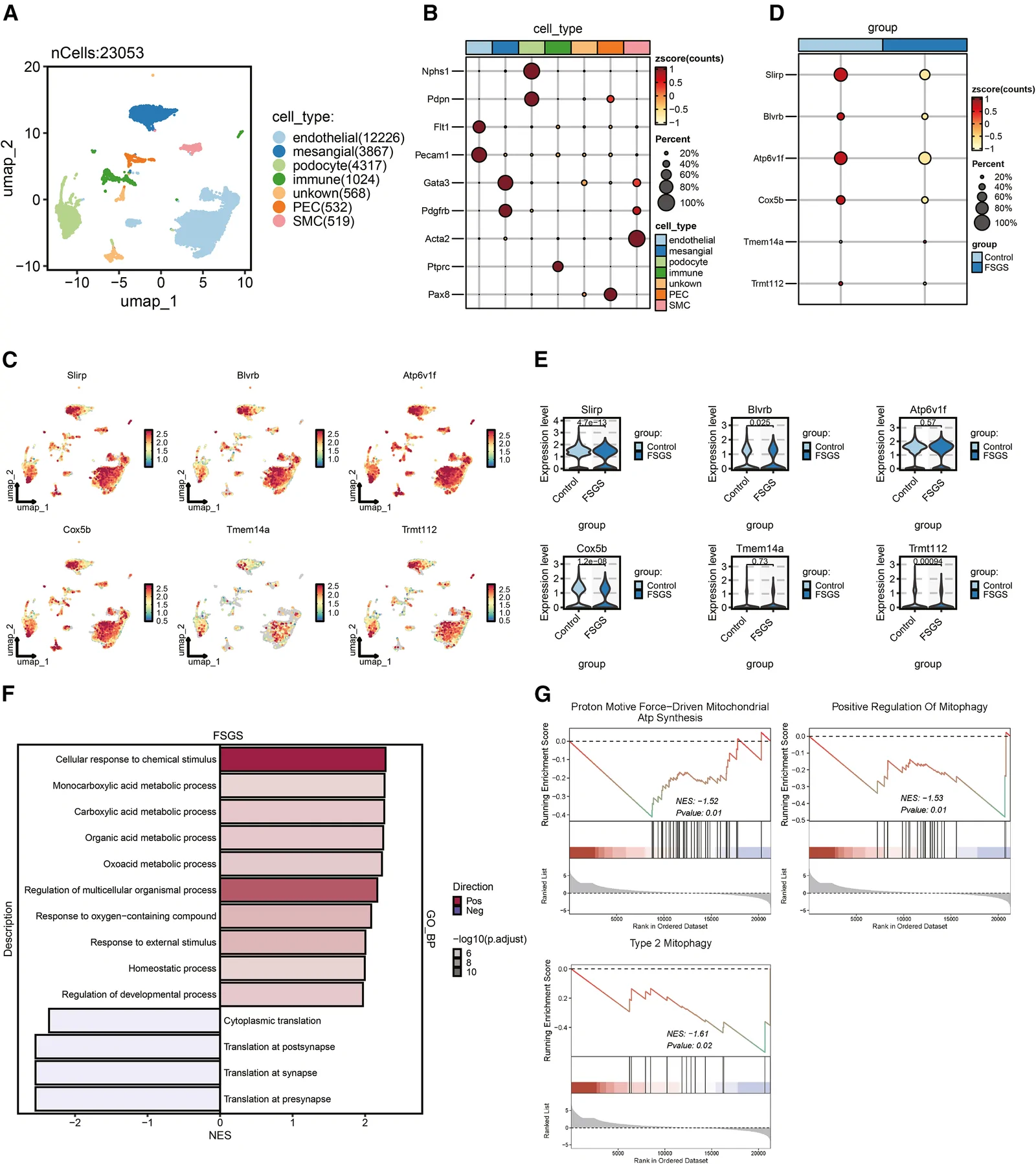
Single-cell transcriptomic analysis identifies podocyte-associated transcriptomic alterations and core pathological mechanisms in the adriamycin-induced mouse FSGS model (A) UMAP visualization of 23,053 high-quality kidney cells from normal control and adriamycin-induced FSGS mice, colored by 7 annotated cell types. (B) Dot plot showing canonical marker gene expression across all annotated kidney cell populations. (C) Feature plots visualizing the expression profile of six core hub genes across all renal cell types, validating their stable expression in podocytes. (D) Dot plot comparing the expression of the six candidate genes between the control and FSGS groups. Color intensity represents the *Z* score normalized average expression, and dot size indicates the percentage of cells expressing the gene. (E) Violin plots quantifying the expression levels of the six genes in podocytes from the control and FSGS groups (scRNA-seq dataset: 3 control mouse kidneys, 2 adriamycin-induced FSGS mouse kidneys). Statistical significance was determined by the Wilcoxon rank-sum test. (F) GSEA of GO-BP in podocytes from FSGS. Bars are colored by -log10(*P*-adjusted), and bar length represents the normalized enrichment score (NES). Red bars indicate upregulated biological processes, while blue bars indicate downregulated processes. (G) GSEA plots showing the enrichment status of three key pathways in podocytes from FSGS: Proton Motive Force-Driven Mitochondrial ATP synthesis, Positive Regulation of Mitophagy, and Type 2 Mitophagy.

UMAP dimensionality reduction and cell clustering annotation revealed that the 23,053 high-quality kidney cells after quality control filtering were clearly divided into 7 major cell clusters (Figure 8A). Dot-plot visualization of canonical cell-type marker genes further validated the identity of each annotated cell population (Figure 8B). Cell count statistics showed that these clusters included podocytes (4317 cells), endothelial cells (12,226 cells), mesangial cells (3867 cells), immune cells (1024 cells), parietal epithelial cells (532 cells), smooth muscle cells (519 cells), and unknown cells (568 cells) (Figure 8A), achieving accurate annotation of the main cell types in mouse kidney tissues. Single-cell expression profile analysis demonstrated that the six core hub genes identified in this study (ATP6V1F, BLVRB, COX5B, SLIRP, TMEM14A, TRMT112) were all stably expressed in podocytes, confirming the direct correlation between this gene set and podocyte biological function (Figure 8C). After isolating podocytes, a dot plot comparing gene expression between the healthy control and FSGS groups showed that, except for TMEM14A, the expression levels of the other five genes were lower in the FSGS group (Figure 8D). Differential expression analysis between groups in the podocyte subpopulation showed that the expression levels of SLIRP, BLVRB, COX5B, and TRMT112 were significantly downregulated in podocytes from FSGS model mice compared with the normal control group (*p* < 0.05). Although ATP6V1F and TMEM14A did not reach the threshold of statistically significant difference, they also exhibited a consistent downregulation trend. These scRNA-seq observations show expression trends in podocytes that are broadly consistent with whole-kidney bulk transcriptome results, providing bioinformatic inference for podocyte-associated alterations of this hub-gene set under FSGS pathological conditions (Figure 8E).

GSEA of GO-BP revealed a distinct dichotomous response in podocytes following adriamycin injury. Consistent with adriamycin-induced cytotoxicity, the top-enriched pathways involved cellular stress responses and metabolic adaptation. The “cellular response to chemical stimulus” was the most significantly activated pathway, reflecting the activation of DNA damage responses and oxidative stress defenses. Concurrently, multiple interconnected metabolic pathways, including carboxylic acid, organic acid, and monocarboxylic acid metabolism, were strongly induced. This signature indicates a shift toward glycolytic and alternative energy production pathways to compensate for mitochondrial respiratory chain dysfunction. Additional activated processes included responses to oxygen-containing compounds and the regulation of multicellular/homeostatic processes, underscoring the activation of cytoprotective and adaptive mechanisms (Figure 8F). Further GSEA analysis highlighted a profound failure in mitochondrial homeostasis (Figure 8G). Pathways governing “Proton Motive Force-Driven Mitochondrial ATP Synthesis” were significantly downregulated (NES = −1.52, *p* < 0.05), confirming a collapse in oxidative phosphorylation capacity. Concomitantly, pathways regulating mitophagy were suppressed, including “Type 2 Mitophagy” (NES = −1.61, *p* < 0.05) and “Positive Regulation of Mitophagy” (NES = −1.53, *p* < 0.05). This dual defect-the inability to generate energy coupled with the failure to clear damaged organelles-creates a vicious cycle of cellular damage, driving the progression to FSGS.

### Validation of candidate genes

To further validate the expression levels of the six candidate genes, we established an FSGS mouse model via a single tail vein injection of adriamycin. On day 35, mice were euthanized, and serum was collected to measure blood creatinine levels, while kidney tissues underwent PAS staining. Compared to controls, the adriamycin group exhibited significantly elevated blood creatinine levels (Figure 9A). PAS staining revealed glomerular segmental sclerosis, accompanied by basement membrane thickening, mesangial matrix expansion, and tubular atrophy in the FSGS group (Figure 9B). Quantitative real-time PCR analysis of kidney tissues showed significant downregulation of SLIRP, BLVRB, COX5B, TMEM14A, and TRMT112 mRNA expression in the FSGS group (Figure 9C). Notably, ATP6V1F expression displayed a downward trend in the FSGS group, but the difference was not statistically significant. Western blot analysis was performed using mouse renal tissues to detect the protein expression of the six candidate genes. Consistent with the qRT-PCR trend, SLIRP, BLVRB, COX5B, TMEM14A, and TRMT112 were significantly downregulated at the protein level in FSGS kidney tissues (*p* < 0.05). Similarly, ATP6V1F also showed statistically significant downregulation at the protein level (*p* < 0.05), even though its mRNA expression did not reach statistical significance (Figure 9D). It should be noted that these mRNA and protein validations represent tissue-level observations and do not denote cell-type-specific alterations. Taken together, these transcriptional and translational observations reveal altered expression of the six-gene signature in FSGS renal tissues and provide supportive evidence for the decreased expression tendency of these candidates found in human transcriptomic datasets.

**Figure 9:**
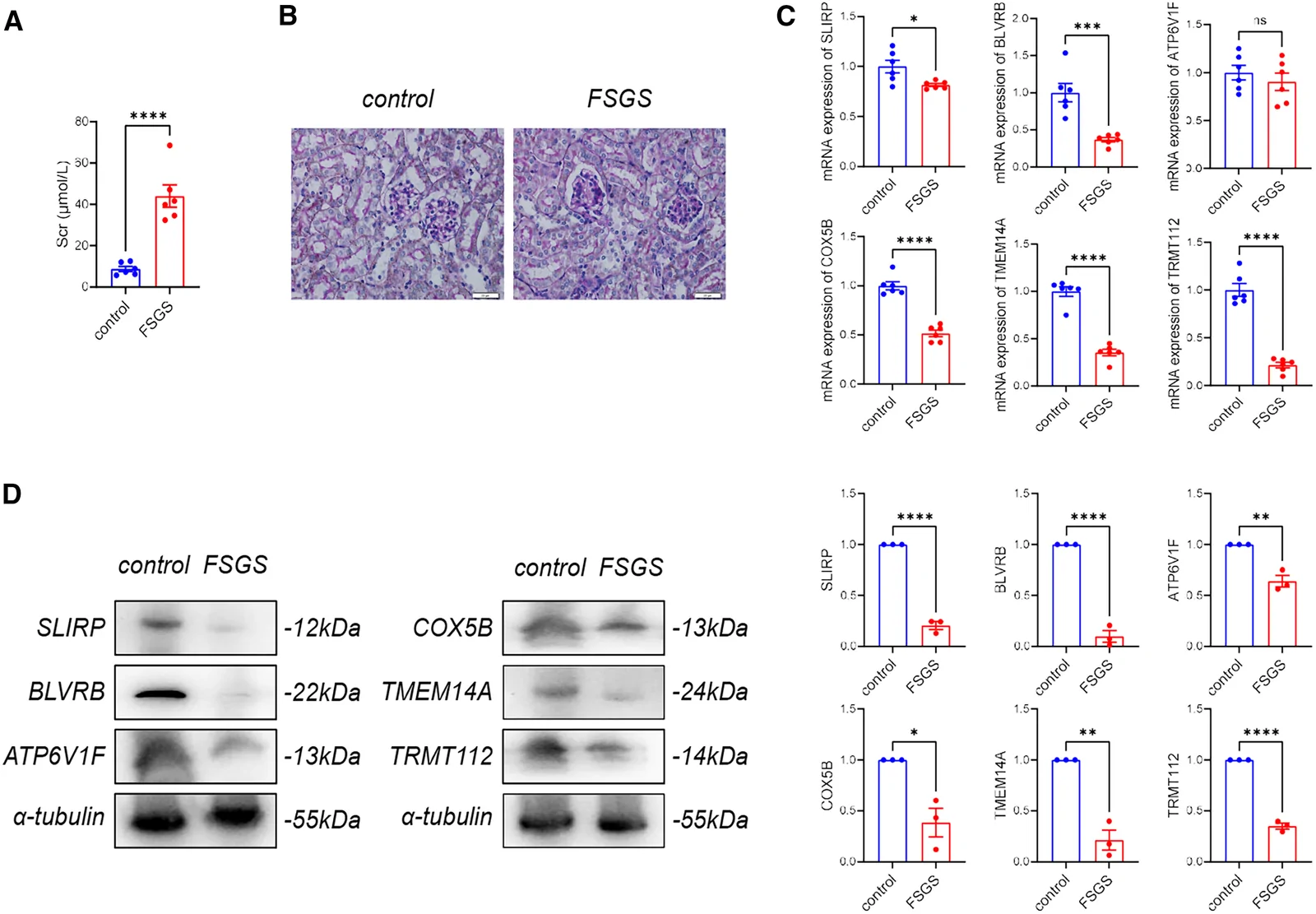
Differential analysis of candidate genes in animal models (A) Serum creatinine (SCr) levels in control and FSGS groups. (B) Representative PAS staining of mouse kidney tissue from the control and FSGS groups at 400× magnification; scale bars, 20 μm. (C) qRT-PCR analysis of six candidate genes in mouse kidney tissue from control and FSGS groups (*n* = 6). (D) Representative western blot images and densitometric quantification of six candidate proteins in mouse renal tissues from control and FSGS groups (*n* = 3). Band intensity was normalized to the internal loading control α-tubulin. Data were represented as mean ± SEM and analyzed using the unpaired two-tailed Student’s *t* test. ∗*p* < 0.05, ∗∗*p* < 0.01, ∗∗∗*p* < 0.001, and ∗∗∗∗*p* < 0.0001; ns, no significance.

## Discussion

This study integrated two GEO transcriptomic datasets, identifying 839 DEGs (276 upregulated, 563 downregulated) through differential expression analysis. WGCNA revealed 212 FSGS-related module genes, and their intersection with DEGs and 5,150 mitophagy-related genes from GeneCards yielded 33 DMRGs. GO and KEGG analyses indicated DMRG involvement in translation, mitochondrial function, protein binding, RNA binding, ribosomal structure, and pathways such as oxidative phosphorylation. Using machine learning algorithms (RF-RFE and SVM-RFE), we identified six core candidate genes: SLIRP, BLVRB, ATP6V1F, COX5B, TMEM14A, and TRMT112 as key FSGS markers. Given the limited initial sample size, we performed multi-dataset large-sample validation and confirmed that the high diagnostic efficacy of this gene signature was not caused by overfitting or random bias. We further used the xCell algorithm to assess renal immune infiltration and identified marked immune microenvironment dysregulation in FSGS, with the six hub genes closely correlated with multiple immune cell subsets. Multi-cohort correlation analysis combined with GSVA and co-expression-based GSEA further verified the intrinsic association of this gene signature with mitophagy defects and mitochondrial dysfunction. ROC analysis and nomogram modeling confirmed the generally satisfactory diagnostic performance of this mitochondrial gene signature. Single-cell transcriptomics further suggested the podocyte-associated expression trends for these hub genes and uncovered dysregulated mitochondrial energy metabolism and mitophagy-related pathways. Subsequent qRT-PCR and western blot further confirmed expression changes of these genes at the renal-tissue level in an FSGS mouse model.

We further compared our six candidate genes with previously reported transcriptomic biomarker signatures in FSGS. Earlier transcriptome studies have established multiple gene panels closely implicated in FSGS pathogenesis, which are mainly involved in renal fibrosis, inflammatory responses, and general podocyte injury.^18^^,^^19^^,^^20^ In contrast, the candidate genes identified in the present study are functionally annotated in mitochondrial homeostasis and mitophagy regulation, a research direction that remains relatively understudied in FSGS. Importantly, none of our six hub genes overlapped with previously established FSGS transcriptomic signatures, demonstrating that our mitophagy-associated gene panel provides additional complementary biomarker resources for FSGS research.

Although these six genes were retrieved from a mitophagy-associated gene set, they are mainly annotated in fundamental mitochondrial processes, including oxidative phosphorylation, mitochondrial translation, redox homeostasis, and lysosomal acidification-all of which are essential for mitochondrial quality control. The coordinated downregulation of these genes in FSGS kidneys suggests a potential association with impaired mitochondrial homeostasis and podocyte injury, but their causal role in mitophagy regulation remains to be functionally validated. SLIRP, a mitochondrial RNA-binding protein, stabilizes mRNAs and regulates translation via the LRPPRC-SLIRP complex, supporting oxidative phosphorylation critical for podocyte energy demands.^21^ Its dysregulation may disrupt mitochondrial function and contribute to podocyte injury.^21^ SLIRP also modulates antiviral signaling in autoimmune diseases and nuclear receptor signaling in cancer, suggesting broader immune and metabolic impacts.^22^^,^^23^ BLVRB, a cytosolic enzyme, reduces biliverdin to bilirubin, contributing to heme catabolism and antioxidant defense.^24^ Its downregulation in FSGS may impair redox homeostasis, increasing oxidative stress and podocyte damage.^25^^,^^26^ ATP6V1F, a subunit of vacuolar H^+^- ATPase, is essential for lysosomal acidification and autophagy, processes vital for podocyte homeostasis.^27^ Its dysregulation, linked to neurodegenerative diseases and cancer, may disrupt lysosomal function, contributing to protein accumulation and cellular stress.^28^^,^^29^ COX5B, a cytochrome *c* oxidase subunit, supports mitochondrial respiration, and its downregulation impairs energy production, potentially contributing to podocyte apoptosis.^30^ Similar disruptions are noted in mitochondrial disorders and cancer.^31^^,^^32^ TMEM14A, a mitochondrial membrane protein, regulates heme biosynthesis, and its dysregulation may induce oxidative stress and mitochondrial dysfunction, as seen in neurodegenerative and oncologic contexts.^33^^,^^34^ TRMT112, a cofactor for tRNA/rRNA methyltransferases, ensures RNA stability and protein synthesis, and its downregulation may impair podocyte function, mirroring effects in mitochondrial and neurodevelopmental diseases.^35^^,^^36^ Although direct experimental evidence validating the functions of these six genes in FSGS remains scarce, they collectively preserve podocyte mitochondrial homeostasis under physiological conditions; their coordinated downregulation in diseased kidneys exacerbates mitochondrial injury and subsequent podocyte dysfunction, strongly implying their pathogenic participation in FSGS.

The infiltration level of immune cells has been reported to influence the development of FSGS, and our study confirmed a significant immune infiltration imbalance in FSGS, characterized by enriched pro-inflammatory effector immune cells and decreased naive and regulatory immune subsets. Mechanistically, active CD8 T cells exacerbate FSGS by releasing miR-186-5p-enriched exosomes, activating TLR7/8 signaling and renal inflammation, with depletion reducing injury in mouse models.^37^ Naive CD4 T cells differentiate into proinflammatory Th1 and Th17 subsets, producing IFN-γ and IL-17 to promote podocyte damage.^38^^,^^39^ Consistent with these findings, our results revealed prominent depletion of naive CD4 and CD8 T cells alongside significant accumulation of CD4 and CD8 Tem cells in FSGS lesions, indicating extensive activation and pro-inflammatory transformation of naive T cells in diseased renal tissues. Moreover, elevated infiltration of NKT cells, eosinophils, macrophages, M2 macrophages, and Tgd cells further sustains local inflammatory cascades and exacerbates glomerular damage.^40^^,^^41^ Importantly, reduced Treg infiltration in FSGS further explains the breakdown of renal immune homeostasis and immune tolerance-the deficiency of these anti-inflammatory regulatory cells fails to constrain local inflammatory responses, ultimately leading to uncontrolled renal inflammation.^40^^,^^42^ Combined with decreased neutrophil abundance, these results demonstrate that FSGS presents heterogeneous immune dysregulation rather than simple pan-inflammatory activation.

On the basis of the above immune landscape changes, our study identified significant correlations between the six mitochondrial hub genes and renal immune infiltration, showing correlative trends that hint at possible crosstalk between hub gene dysregulation and FSGS immune dysregulation. Most gene-immune correlations were consistent with the FSGS inflammatory phenotype. The downregulated hub genes were negatively correlated with FSGS-enriched pro-inflammatory subsets, including macrophages, CD8 Tem cells, eosinophils, and Tgd cells. Mechanistically, dysregulated TMEM14A (heme biosynthesis^33^) and TRMT112 (RNA stability^35^) trigger mitochondrial stress; ATP6V1F deficiency impairs lysosomal autophagy and mitochondrial clearance,^27^ thereby activating pro-inflammatory TLR7/8 signaling.^37^ Downregulated BLVRB disrupts redox balance via altered biliverdin metabolism,^24^^,^^25^^,^^26^ aggravating CD4 T cell-mediated Th1/Th17 oxidative stress and podocyte injury,^38^^,^^39^ while reduced COX5B expression impairs mitochondrial respiration.^30^ Collectively, these abnormalities disrupt immune homeostasis and amplify renal inflammation. Furthermore, these hub genes were positively correlated with FSGS-depleted neutrophils and naive CD8 T cells. Their downregulation disturbs mitochondrial autophagy, energy metabolism, and RNA modification,^27^^,^^30^^,^^33^^,^^35^ impairing protective immune cell homeostasis and further disrupting renal immune balance. Notably, only the Treg correlation showed an inconsistent trend. ATP6V1F and BLVRB were negatively correlated with Tregs; their downregulation was predicted to increase Treg infiltration, whereas Treg depletion was observed in FSGS lesions. This discrepancy indicates that the FSGS pathological microenvironment may override intrinsic gene-immune regulatory patterns, causing impaired immune tolerance and persistent renal inflammation. Collectively, these findings indicate a potential association between the transcriptomic downregulation of mitochondrial hub gene dysfunction and FSGS immune dysregulation, and further functional experiments are required to clarify whether there is a direct regulatory or causal relationship.

These DMRGs are also involved in various molecular signaling pathways, such as translation, structural composition of mitochondria and ribosomes, and protein and RNA binding, which affect the occurrence and development of FSGS. Altered expression of these DMRGs is correlated with transcriptomic changes of mitochondrial homeostasis pathways, which may be linked to energy deficits, oxidative stress, and podocyte injury in FSGS.^1^ For instance, SLIRP’s role in mitochondrial translation is critical for podocyte energy production, and its downregulation disrupts oxidative phosphorylation.^21^ Similarly, DMRGs encoding ribosomal or RNA-binding proteins affect mitochondrial protein synthesis, linked to podocyte apoptosis in FSGS.^38^ Protein-binding pathways, particularly cytoskeletal regulators, contribute to foot process effacement and proteinuria, as seen in FSGS and diabetic nephropathy.^1^^,^^43^ Immune-mediated inflammation, amplified by translation-dependent cytokine production, further drives FSGS progression, with elevated proinflammatory cytokines observed in patients.^38^ These findings highlight DMRGs’ multifaceted roles in FSGS pathogenesis, providing potential research candidates for exploring mitochondrial homeostasis and mitophagy in FSGS.

In this study, single-cell transcriptomic analysis further indicated the podocyte-associated expression trends for the six hub genes. Most of these mitochondrial genes were significantly downregulated in FSGS podocytes, which was largely consistent with bulk transcriptome results. Intriguingly, several genes only presented a marginal downward trend without statistical significance at both single-cell and animal model levels, especially ATP6V1F. Such subtle expression divergence may be attributable to interspecies variation, discrepant pathological stages between clinical specimens and experimental models, as well as individual sample heterogeneity. Functionally, injured FSGS podocytes activated cellular stress responses and metabolic adaptation, while presenting remarkable suppression of mitochondrial oxidative phosphorylation and mitophagy-related pathways. This dual defect of insufficient energy supply and impaired clearance of damaged organelles exacerbates mitochondrial dysfunction and accelerates progressive podocyte injury. These single-cell findings reveal transcriptomic signatures suggestive of disturbed mitochondrial quality control in podocytes under FSGS pathology, reinforcing the biological relevance of the identified hub gene signature.

Nevertheless, mitophagy participates in FSGS pathogenesis, and this study integrated transcriptomic data, WGCNA, and machine learning to screen mitophagy-related genes and identified six candidate hub genes (SLIRP, BLVRB, ATP6V1F, COX5B, TMEM14A, and TRMT112). Multi-cohort transcriptomic analyses confirmed strong correlations of this signature with transcriptomic alterations of mitophagy and mitochondrial function-related pathways. ROC and nomogram analyses revealed generally acceptable discriminatory capacity for this gene signature across independent cohorts, with moderate efficacy fluctuations between datasets, and the signature is closely correlated with renal immune dysregulation in FSGS. Single-cell transcriptomics suggested a trend of downregulation of these genes in FSGS podocytes, together with concurrent suppression of mitochondrial energy metabolism and mitophagy-related pathways. These expression alterations were further verified at the whole-renal-tissue level in adriamycin-induced mouse models. Collectively, our findings reveal correlative trends suggesting a possible association between hub gene downregulation, transcriptomic alterations of podocyte mitochondrial pathways, and renal immune imbalance, which may provide candidate biomarkers and research clues for mitochondrial mechanism studies in FSGS.

### Limitations of the study

Despite comprehensive analyses, this study has several notable limitations. Bulk and single-cell transcriptomic datasets suffer from limited sample sizes alongside incomplete clinical metadata, which reduces sample diversity. All human transcriptomic data were obtained from retrospective, heterogeneous public GEO databases; these data characteristics may lead to moderate AUC performance in certain external validation cohorts and introduce intrinsic selection bias. Moreover, although we verified gene expression at both the mRNA and protein levels in mouse kidney tissues, ATP6V1F showed inconsistent expression trends between qRT-PCR and western blot, which may be attributed to complex post-transcriptional regulation. Third, our mRNA and protein-level experimental validations were performed using whole-kidney tissue lysates from mice, which only reflect overall gene expression in renal tissue and cannot establish podocyte-specific expression or biological functions for these mitophagy-associated candidate genes. In addition, all mitophagy-related conclusions in this work are derived from bioinformatic analyses of public transcriptomic datasets. Direct experimental evidence, including mitophagy flux detection, assessment of mitochondrial morphology, reactive oxygen species levels, mitochondrial membrane potential measurement in podocytes, as well as gene knockout/overexpression functional assays, is still lacking. Collectively, our mRNA and protein data merely demonstrate correlations rather than causal relationships or direct mechanistic connections with mitophagy. Further cell-specific experimental validations such as immunofluorescence co-staining are also required. xCell immune deconvolution based on bulk RNA-seq is exploratory, and the observed gene-immune correlations cannot validate direct regulatory interactions without functional assays. Future studies should leverage larger FSGS cohorts, integrate multi-omics data, and conduct functional experiments (e.g., in adriamycin models) including multi-timepoint animal validation to clarify the dynamic expression patterns and molecular mechanisms of these candidate genes during FSGS progression.

## Resource availability

### Lead contact

Further information and requests for resources should be directed to and will be fulfilled by the corresponding author, Haijian Wei (weihaijian88@126.com).

### Materials availability

This study did not generate new unique reagents or biological materials.

### Data and code availability

• Data: All publicly available gene expression and single-cell datasets analyzed in this study were retrieved from the NCBI Gene Expression Omnibus (GEO) under the following accession numbers: GEO: GSE125779, GEO: GSE129973, GEO: GSE108109, GEO: GSE108112, GEO: GSE200818, GEO: GSE200828, GEO: GSE104948, GEO: GSE104954, and GEO: GSE146912. These datasets are publicly accessible without restrictions, material transfer agreements, or additional authorization. All data generated or analyzed during this study are included in the manuscript and its supplementary materials. Data reported in this paper will be shared by the lead contact upon request.
• Code: This paper does not report original code. All bioinformatic analyses were performed using standard, publicly available R packages with specified versions, as described in the STAR Methods section. All workflows are fully reproducible using the parameters provided in the manuscript.
• Other items: Any additional information required to reanalyze the data reported in this paper are available from the lead contact upon request.

## Acknowledgments

This work was funded by the Yantai Science and Technology Innovation Development Plan (Grant No. 2025YD081) and the 10.13039/501100007129Shandong Provincial Natural Science Foundation (Youth Project, Grant No. ZR2026QC2175Z). The Graphical Abstract was created with BioRender (biorender.com).

## Author contributions

J.M.: writing - original draft, writing - review and editing, formal analysis, investigation, and funding acquisition; T.M.: writing - original draft, methodology, formal analysis, and investigation; Y.Z.: conceptualization, methodology, and supervision; H.W.: writing - review and editing, conceptualization, supervision, funding acquisition, and resources.

## Declaration of interests

The authors declare no competing interests.

## STAR★Methods

### Key resources table

**Table:** 

REAGENT or RESOURCE	SOURCE	IDENTIFIER
**Antibodies**		

Rabbit polyclonal anti-SLIRP	Invitrogen	Cat#PA5-115923; RRID: AB_2900557
Rabbit polyclonal anti-BLVRB	Invitrogen	Cat#PA5-96619; RRID: AB_2808421
Rabbit monoclonal anti-ATP6V1F	Abcam	Cat#ab190789; RRID: AB_2892713
Rabbit monoclonal anti-COX5B	Abcam	Cat#ab110263; RRID: AB_10860497
Rabbit polyclonal anti-TMEM14A	Novus	Cat#NBP2-56394; RRID: AB_3341537
Rabbit polyclonal anti-TRMT112	Invitrogen	Cat#PA5-120266; RRID: AB_2913838
Mouse monoclonal anti-α-tubulin	Abcam	Cat#ab7291; RRID: AB_2241126
HRP-conjugated anti-rabbit IgG secondary antibody	Cell Signaling Technology	Cat#7074; RRID: AB_2099233
HRP-conjugated anti-mouse IgG secondary antibody	Cell Signaling Technology	Cat#7076; RRID: AB_330924

**Chemicals, peptides, and recombinant proteins**		

Adriamycin (Doxorubicin)	Sigma-Aldrich	Cat#D1515
Isoflurane	MedChemExpress	Cat#HY-A0134
RIPA lysis buffer	Beyotime	Cat#P0013B
Protease inhibitor cocktail	MedChemExpress	Cat#HY-K0010
iBright Prestained Protein Ladder	Invitrogen	Cat#LC5615
10% neutral buffered formalin	Sigma-Aldrich	Cat#HT501128

**Critical commercial assays**		

Creatinine Assay Kit	Nanjing Jiancheng Bioengineering Institute	Cat#C011-2
TRIzol reagent	Thermo Fisher	Cat#15596026CN
HiScript II Q RT SuperMix	Novizan	Cat#R223-01
ChamQ Universal SYBR qPCR Master Mix	Novizan	Cat#Q711-03
BCA Protein Assay Kit	Thermo Fisher	Cat#A65453
Pierce ECL Western Blotting Substrate	Thermo Scientific	Cat#32209

**Deposited data**		

Bulk transcriptome RNA-seq dataset GSE125779	Gene Expression Omnibus, https://www.ncbi.nlm.nih.gov/geo/	GEO: GSE125779
Bulk transcriptome RNA-seq dataset GSE129973	Gene Expression Omnibus, https://www.ncbi.nlm.nih.gov/geo/	GEO: GSE129973
Bulk transcriptome RNA-seq dataset GSE108109	Gene Expression Omnibus, https://www.ncbi.nlm.nih.gov/geo/	GEO: GSE108109
Bulk transcriptome RNA-seq dataset GSE108112	Gene Expression Omnibus, https://www.ncbi.nlm.nih.gov/geo/	GEO: GSE108112
Bulk transcriptome RNA-seq dataset GSE200818	Gene Expression Omnibus, https://www.ncbi.nlm.nih.gov/geo/	GEO: GSE200818
Bulk transcriptome RNA-seq dataset GSE200828	Gene Expression Omnibus, https://www.ncbi.nlm.nih.gov/geo/	GEO: GSE200828
Bulk transcriptome RNA-seq dataset GSE104948	Gene Expression Omnibus, https://www.ncbi.nlm.nih.gov/geo/	GEO: GSE104948
Bulk transcriptome RNA-seq dataset GSE104954	Gene Expression Omnibus, https://www.ncbi.nlm.nih.gov/geo/	GEO: GSE104954
Single-cell RNA-seq dataset GSE146912	Gene Expression Omnibus, https://www.ncbi.nlm.nih.gov/geo/	GEO: GSE146912
Mitophagy-related gene list	GeneCards database, https://www.genecards.org/	accessed May 14, 2024; see Table S1 (Data S1)

**Experimental models: Organisms/strains**		

Mouse: BALB/c; SPF male mice	Charles River Laboratories (Beijing, China)	N/A

**Oligonucleotides**		

BLVRB-F 5′-ATGCCCCCACACATAGGAGA-3′	ComateBio	N/A
BLVRB-R 5′-TCCCCACCTAGGTCAGAGTG-3′	ComateBio	N/A
SLIRP-F 5′-TAAAGGGGCTAAGCGCACTG-3′	ComateBio	N/A
SLIRP-R 5′-TGTTCTCTCAGCTCACTCGC-3′	ComateBio	N/A
COX5B-F 5′-TAGACTCCCACCAACGGAAG-3′	ComateBio	N/A
COX5B-R 5′-GCATATTGTATGGGTCCAGTCC-3′	ComateBio	N/A
ATP6V1F-F 5′-GATCGAAGACACTTTCAGGCAAT-3′	ComateBio	N/A
ATP6V1F-R 5′-CTTTGGCTGCATCGTAGGGA-3′	ComateBio	N/A
TRMT112-F 5′-GGCAGCGGACACCTTAAACT-3′	ComateBio	N/A
TRMT112-R 5′-CGGCTAGCAAGGTTACGTCT-3′	ComateBio	N/A
TMEM14A-F 5′-CGACTTCACTTCCCAGGGTC-3′	ComateBio	N/A
TMEM14A-R 5′-TGGTAAGGATGCAAGTCTGC-3′	ComateBio	N/A
β-actin-F 5′-TTCAACACCCCAGCCATG-3′	ComateBio	N/A
β-actin-R 5′-CCTCGTAGATGGGCACAGT-3′	ComateBio	N/A

**Software and algorithms**		

R (v4.3.3)	R Foundation	https://www.r-project.org
R package GEOquery	Davis and Meltzer^44^	Bioconductor
R package sva	Leek et al.^45^	Bioconductor
R package limma	Ritchie et al.^46^	Bioconductor
R package ggplot2	Wickham^47^	CRAN
R package WGCNA	Langfelder and Horvath^48^	CRAN
R package VennDiagram	Chen and Boutros^49^	CRAN
R package randomForest	Liaw and Wiener^50^	CRAN
R package e1071	Meyer et al.	CRAN
R package caret	Kuhn^51^	CRAN
R package rms	Harrell	CRAN
R package ComplexHeatmap	Gu et al.^52^	Bioconductor
R package pROC	Robin et al.^53^	CRAN
Seurat (v5)	Hao et al.^54^	https://satijalab.org/seurat/
R package SCOP	Meng Xu et al.	CRAN
DAVID functional annotation tool	NIH	https://davidbioinformatics.nih.gov
GraphPad Prism	GraphPad Software	Version 8.0.1
ImageJ	Schneider et al.^55^	https://imagej.net/ij/

### Experimental model and study participant details

#### Human-derived public transcriptomic data

No human participants or original human biospecimens were collected in this study. All human-related analyses were performed using de-identified archived datasets downloaded from the GEO repository. Original sample collection and ethical review for these deposited datasets were completed by the respective original investigators. No additional human-ethics approval or informed-consent procedures were required for our secondary bioinformatic re-analysis of publicly available datasets. As no human subjects were directly enrolled, no allocation of human subjects to experimental groups was performed.

#### Mouse experimental model

Specific pathogen-free (SPF) wild-type 8-week-old male BALB/c mice with an average body weight of 20–22 g were used for *in-vivo* experiments. Mice were acclimatized for one week under controlled SPF conditions (12-h light/dark cycle, 22 ± 2°C, 50 ± 10% humidity, standard chow, and water *ad libitum*) at the animal facility of the First Hospital of Jilin University. Mice were randomly assigned to FSGS and control groups using a random number generator (*n* = 6 per group). Because only male mice were used, the potential sex-specific influence on the experimental outcomes was not separately assessed; this is consistent with the conventional use of male mice in this disease model. All procedures were approved by the Animal Ethics Committee of the First Hospital of Jilin University (Approval Number: 20240347) and performed in accordance with institutional animal welfare regulations and the ARRIVE guidelines.

#### Cell lines

No cell lines were used in this study.

### Method details

#### Data collection

mRNA expression datasets (GEO: GSE125779, GEO: GSE129973, GEO: GSE108109, GEO: GSE108112, GEO: GSE200818, GEO: GSE200828, GEO: GSE104948, and GEO: GSE104954) were retrieved from the Gene Expression Omnibus (GEO, http://www.ncbi.nlm.nih.gov/geo/). GSE125779 and GSE129973 were merged as the primary dataset. GSE108109, GSE108112, GSE200818, and GSE200828 were integrated with the primary dataset for large-sample robustness validation and overfitting assessment of machine learning models, while GSE104948 and GSE104954 served as external validation datasets (Table 1). Additionally, the scRNA-seq dataset GEO: GSE146912 was retrieved for subsequent cell-type-specific expression and pathway enrichment analysis (Table 1). Mitophagy-related genes were collected from the GeneCards database (https://www.genecards.org/, accessed on May 14, 2024) using “mitophagy” as the keyword. Since mitophagy is a key process in mitochondrial quality control, this approach was used to comprehensively identify genes associated with mitochondrial function and homeostasis, without overly strict pathway restriction to ensure screening comprehensiveness. This search retrieved a total of 5,150 genes, which are listed in Table S1. The relevance scores for each gene are also provided in this supplementary table.

#### Screening of differentially expressed genes

The R package “GEOquery” was used for data retrieval and probe annotation. Batch effects between GSE125779 and GSE129973 were removed using the “sva” package, followed by dataset merging. Principal component analysis (PCA) was conducted using the “prcomp” function on both the pre- and post-normalized datasets to assess batch effect removal and to visualize sample variability and clustering patterns. The “limma” package performed differential expression analysis between control and FSGS samples, identifying DEGs with thresholds of |log2 fold change (FC)| > 0.5 and *P*-adjusted <0.05 (log FC > 0.5 with *P*-adjusted <0.05 for upregulated, log FC < −0.5 with *P*-adjusted <0.05 for downregulated DEGs). Volcano plots and heatmaps were generated using the “ggplot2” package.

#### Weighted gene co-expression network analysis

WGCNA was conducted using the top 25% genes by variance analysis from the merged gene expression matrix. Preprocessing involved constructing a sample clustering tree to remove outliers. The optimal soft threshold (β) was selected to ensure a scale-free topology (R^2^ > 0.9), and a topological overlap matrix (TOM) was built to partition the gene expression matrix into modules (minimum 50 genes per module). Module-trait correlations were calculated, and the module most correlated with FSGS was selected as the key module. Gene significance (GS) and module membership (MM) were assessed for module genes. DEGs, WGCNA module genes, and mitophagy-related genes were intersected using the “VennDiagram” package to identify DMRGs.

#### Functional enrichment analysis

DMRGs’ biological roles were analyzed via the DAVID platform (https://david.ncifcrf.gov/), performing GO annotation including biological process (BP), molecular function (MF), cellular component (CC), and KEGG pathway enrichment. Results were visualized using the “ggplot2” package, with *p* < 0.05 considered statistically significant.

#### Screening candidate genes by RF-RFE and SVM-RFE

Random forest (RF) and support vector machine (SVM) algorithms, combined with recursive feature elimination (RFE), were used to screen DMRGs. The merged dataset was split 7:3 into training and testing sets. RF analysis was performed using the “randomForest” package, SVM using “e1071,” and RFE using “caret”. Intersecting genes from RF-RFE and SVM-RFE were designated as candidate genes using “VennDiagram”.

For robustness evaluation and overfitting assessment, six FSGS transcriptome datasets, including GSE125779, GSE129973, GSE108109, GSE108112, GSE200818, and GSE200828, were integrated for large-sample validation. Batch effects were corrected via the ComBat algorithm, and PCA was applied to verify the effectiveness of batch effect correction. We adopted 100 repetitions 5-fold cross-validation and 1000 label permutation tests to evaluate model discriminative ability and rule out overfitting. Variable importance was further quantified by the mean decrease in Gini coefficient for the RF model and standardized variable weight for the SVM model.

#### Establishment of nomogram model

A nomogram model predicting FSGS risk was constructed using the “rms” package based on candidate genes, visualizing their risk contributions. Candidate gene expression levels in FSGS and control samples were visualized using the “ComplexHeatmap” package.

#### Immune cell infiltration analysis

Using the batch-corrected transcriptome data of 239 kidney samples (50 controls, 189 FSGS), xCell, which covers 64 human cell types, including renal intrinsic stromal and parenchymal cells such as fibroblasts, endothelial cells, mesangial cells, and pericytes,^56^ was applied to calculate enrichment scores of 64 cell types per sample. Wilcoxon rank-sum test with Benjamini-Hochberg false discovery rate (FDR) correction was used to screen differentially infiltrated cell types between groups, with FDR <0.05 as the cutoff. Furthermore, Pearson correlation analysis was performed to assess the correlations between the expression of six core hub genes and cell enrichment scores, and the results were visualized by a heatmap after FDR correction.

#### Validation of candidate genes

Receiver operating characteristic (ROC) analysis, performed with the “pROC” package, quantified candidate gene specificity and sensitivity via area under the curve (AUC) values. Diagnostic value was tested using internal (GSE125779, GSE129973) and external (GSE104948, GSE104954) datasets.

#### Mitophagy-related transcriptome correlation analysis

All analyses were performed on the aforementioned batch-corrected transcriptome data of 239 glomerular samples (50 controls, 189 FSGS). Spearman correlations between six hub genes and canonical mitophagy markers (PINK1, MAP1LC3B, SQSTM1, TOMM20, COX4I1, BNIP3, BNIP3L) were calculated per cohort. All *p* values were adjusted via the Benjamini-Hochberg method. Pooled correlation coefficients across all cohorts were integrated using Fisher’s z transformation to visualize overall correlation patterns in a heatmap. Gene Set Variation Analysis (GSVA) was used to quantify mitophagy activity based on GO_MITOPHAGY and REACTOME_MITOPHAGY gene sets from MSigDB and Reactome. We analyzed the correlation between mitophagy GSVA scores and normalized z-scores of the six-gene signature within each cohort. Genes positively co-expressed with the signature (Spearman’s ρ > 0.5, BH-adjusted *p* < 0.05) were ranked for pre-ranked GSEA. We focused on enrichment entries related to autophagy-lysosome function, mitochondrial structure, membrane potential, and ROS metabolism.

#### Single-cell RNA sequencing data acquisition and processing

The scRNA-seq dataset GSE146912, derived from kidney tissues of adriamycin-induced mouse FSGS models, was downloaded from the GEO database. A total of five samples, including three normal control kidneys (GSM4409507, GSM4409508, GSM4409509) and two adriamycin-induced FSGS kidneys (GSM4409514, GSM4409515), were enrolled for systematic analysis. Raw sequencing data were integrated, normalized, and quality-filtered using Seurat (v5), with high-quality cells retained according to the criteria of 200 < nFeature_RNA <7000 and mitochondrial gene percentage (percent.mt) < 5%, yielding 23,053 qualified cells for subsequent analysis. PCA dimensionality reduction was performed, followed by Uniform Manifold Approximation and Projection (UMAP) visualization and unsupervised clustering based on the top 50 principal components. Cell clusters were annotated using canonical mouse kidney cell markers. The “SCOP” package was used for visualization. The podocyte subpopulation was extracted for targeted transcriptomic analysis: Wilcoxon rank-sum test was applied to compare the expression differences of six core hub genes between the control and FSGS groups (*p* < 0.05 was set as the significance threshold); Gene Set Enrichment Analysis (GSEA) was conducted based on the genome-wide expression profiles of podocytes to identify differential biological pathways between the two groups (*P*-adjusted <0.05 for significant enrichment), with a focus on mitochondrial energy metabolism and mitophagy processes to validate the cell-type-specific alterations observed in our bulk transcriptome analysis.

#### FSGS animal model establishment

The FSGS group received a single tail vein injection of adriamycin (0.01 mg/g body weight) under isoflurane anesthesia (2% induction, 1% maintenance), while the control group received an equivalent volume of saline.

On day 35 post-injection, mice were euthanized via gradual CO_2_ inhalation per national animal welfare guidelines. Blood was collected by cardiac puncture, clotted for 30 min, and centrifuged at 3,000 rpm for 10 min at 4°C to obtain serum for creatinine measurement using a Creatinine Assay Kit. Kidney tissues were harvested, fixed in 10% neutral buffered formalin, and stained with periodic acid-Schiff (PAS). The success of the FSGS model was confirmed by significant elevation in serum creatinine levels and histopathological changes observed in PAS staining.

#### Quantitative real-time PCR

Total RNA was extracted from kidney tissues using Trizol reagent, and its concentration was determined using a NanoDrop spectrophotometer. cDNA was synthesized from 1 μg RNA using HiScript II Q RT SuperMix. Quantitative real-time PCR (qRT-PCR) was conducted using ChamQ Universal SYBR qPCR Master Mix on an Applied Biosystems StepOnePlus system, with target gene mRNA levels calculated using the 2ˆ^−ΔΔCt^ method, normalized to β-actin. Primer sequences are listed in the key resources table.

#### Western blot

For Western blot detection, total protein was extracted from kidney tissues with RIPA lysis buffer containing a protease inhibitor cocktail. Protein concentration was determined using the BCA protein assay. Equal quantities (30 μg per lane) of protein samples were separated on 12% SDS-PAGE gels and transferred to 0.22 μm PVDF membranes. After blocking with 5% skim milk at room temperature for 1 h, membranes were incubated overnight at 4°C with primary antibodies at a dilution of 1:1000, including SLIRP, BLVRB, ATP6V1F, COX5B, TMEM14A, TRMT112, α-tubulin, followed by incubation with HRP-conjugated secondary antibodies (1:5000) at room temperature for 1 h. Protein bands were visualized using Pierce ECL substrate and captured via chemiluminescence imaging. Densitometric quantification of band intensity was performed using ImageJ software, and the relative protein abundance was calculated by normalizing the target protein signal to the corresponding α-tubulin signal on the same blot. All Western blot assays were performed with 3 independent biological replicates per group.

### Quantification and statistical analysis

Investigators were not blinded during allocation and treatment due to a single-investigator design, but blinding was applied during PAS staining and qRT-PCR, and Western blot densitometric quantification to minimize bias.

Statistical analyses were conducted using GraphPad Prism (v8.0.1) and R (v4.3.3). Gene expression differences were compared using the Wilcoxon rank-sum test or unpaired two-tailed Student’s t-tests. The Benjamini-Hochberg FDR method was applied for multiple comparison correction. All tests were two-sided, and a threshold of *p* < 0.05 was considered statistically significant. Significance in the figures is denoted by asterisks: ∗*p* < 0.05, ∗∗*p* < 0.01, ∗∗∗*p* < 0.001, ∗∗∗∗*p* < 0.0001; ns, not significant. Parametric data are shown as the mean ± SEM; non-parametric comparisons use medians. All statistical details of the experiments, including the exact value of n and what n represents (e.g., number of mice or samples), are provided in the corresponding figure legends and Results.

## Supplemental information

Document S1. Figures S1 and S2

Table S1. List of 5,150 mitophagy-related genes and their relevance scores

Data S1. Uncropped and unedited raw western blot images corresponding to Figure 9D
