# Supplementary material for: Identification of mitophagy-related genes with diagnostic value in focal segmental glomerulosclerosis using bioinformatics analysis
Source: iScience. 2026 Oct 23;29(11):118015. doi: 10.1016/j.isci.2026.118015 (PMC13720624; doi:10.1016/j.isci.2026.118015)
Supplement: Supplementary file 1 [file mmc1.pdf]

iScience, Volume *29*

Supplemental information

Identification of mitophagy-related genes with diagnostic value in focal segmental glomerulosclerosis using bioinformatics analysis

Jianan Ma, Tianshuai Ma, Yongrui Zhang, and Haijian Wei

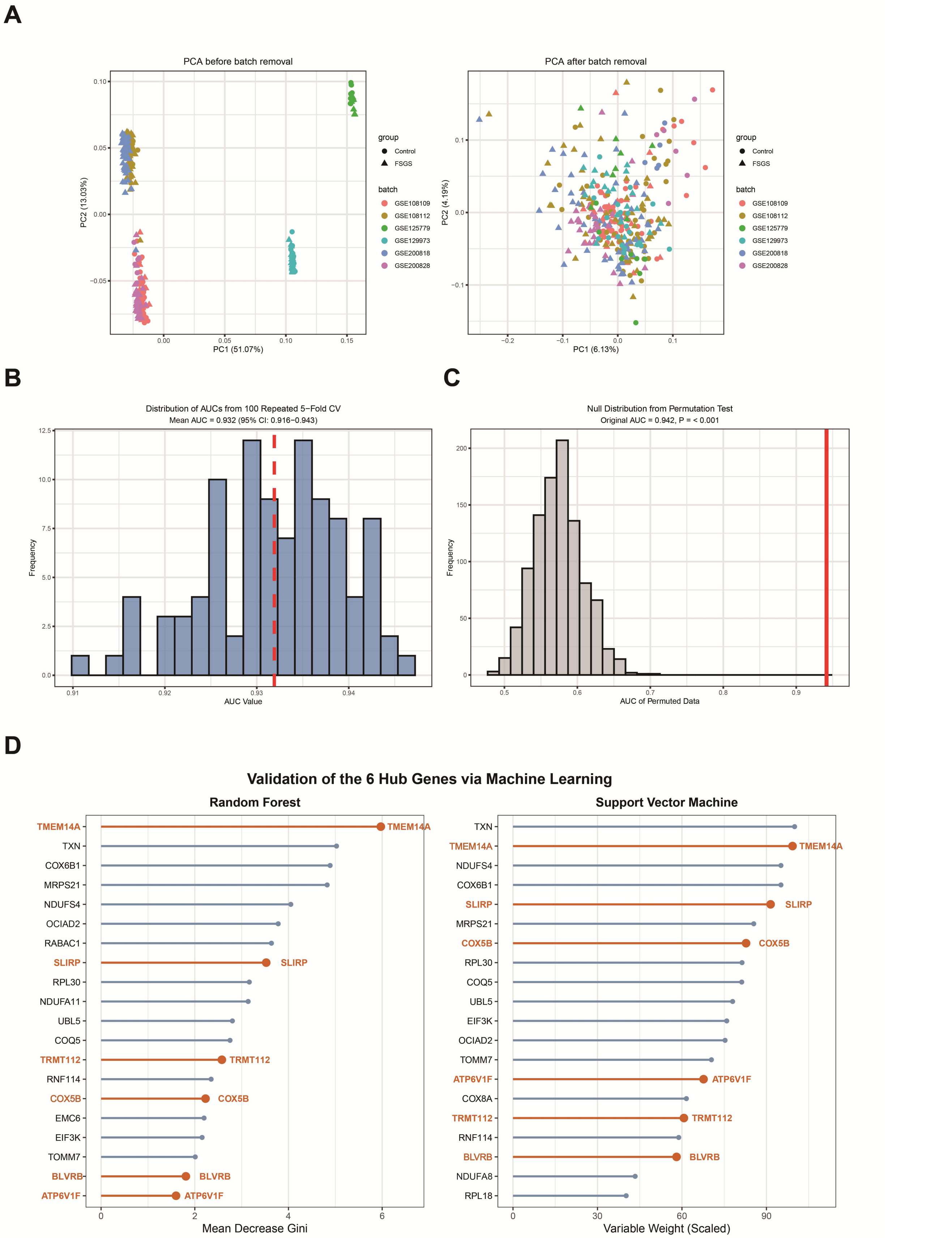

Figure S1 . Validation of six core FSGS-related hub genes in a large-sample multi-dataset cohort.

(A) PCA plots showing sample distribution before (left) and after (right) batch correction using the ComBat algorithm, with samples colored by dataset and group.

(B) Distribution of AUC values obtained from 100 repeated 5-fold cross-validation of the Random Forest model. The mean AUC (0.932, 95% CI: 0.916-0.943) is indicated by the red dashed line.

(C) Null distribution of AUC values from 1000 label permutation tests. The red line indicates the AUC of the original model (0.942, $P < 0.001$).

(D) Variable importance ranking of the six core hub genes in the Random Forest (left) and Support Vector Machine (right) models.

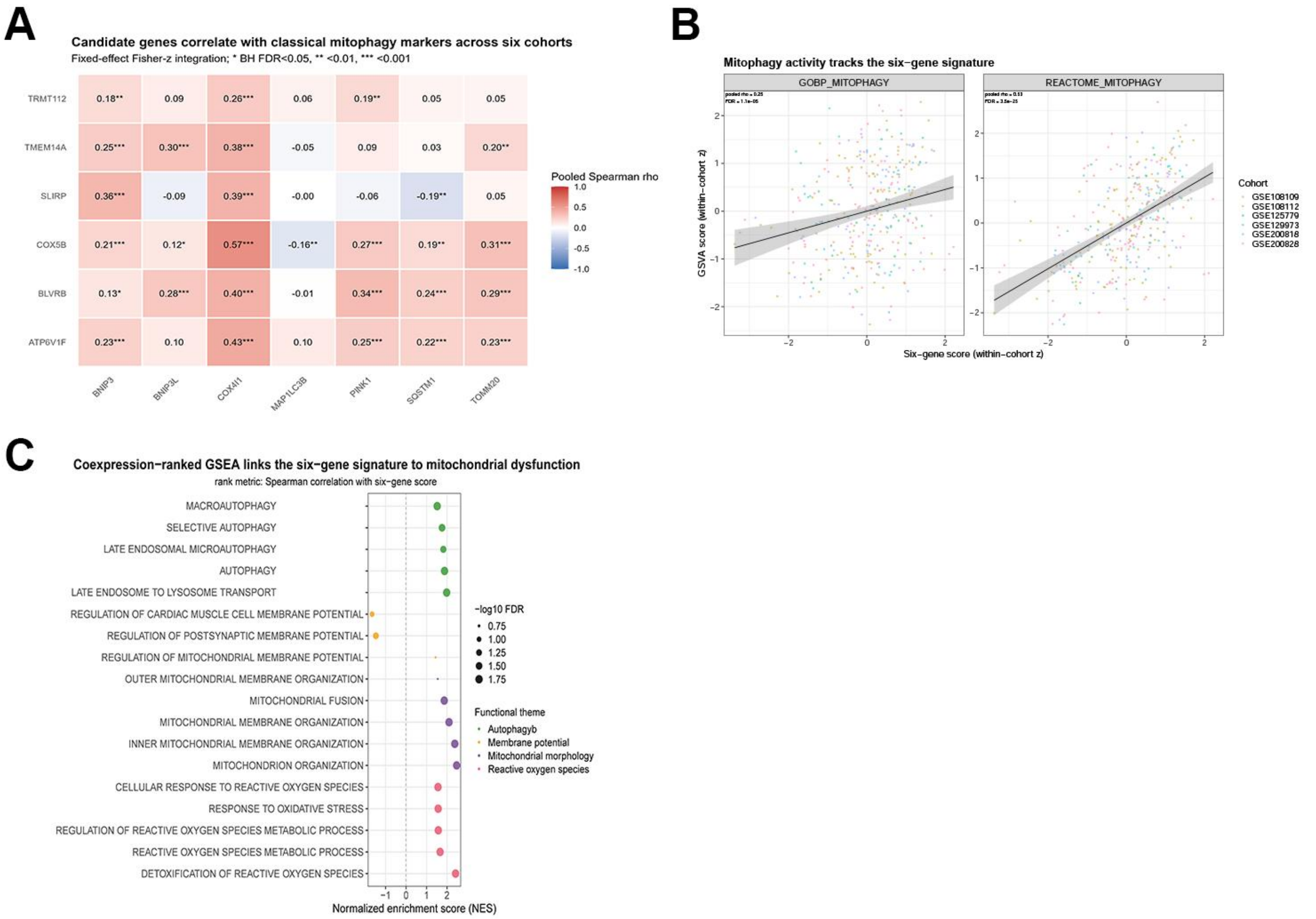

Figure S2. Correlation of the six hub gene signature with mitophagy and mitochondrial dysfunction.

(A) Pooled Spearman correlation heatmap between six hub genes and canonical mitophagy markers across six cohorts. Significance was assessed by BH-adjusted FDR (*P < 0.05, **P < 0.01, ***P < 0.001). Color intensity denotes pooled Spearman rho values.

(B) Scatter plots displaying correlations between six-gene signature z-scores and GSVA scores of two mitophagy gene sets. Data points are grouped by individual GEO cohorts with regression lines and confidence intervals.

(C) GSEA ranked by gene signature correlation. X-axis represents NES. Dot size equals −log10 (FDR), and colors distinguish autophagy, membrane potential, mitochondrial morphology, and ROS-related pathways.
