# Supplementary material for: Identification of mitophagy-related genes with diagnostic value in focal segmental glomerulosclerosis using bioinformatics analysis
Source: iScience. 2026 Oct 23;29(11):118015. doi: 10.1016/j.isci.2026.118015 (PMC13720624; doi:10.1016/j.isci.2026.118015)
Supplement: Supplementary file 2 [file mmc3.pdf]

Data S1. Uncropped and unedited raw western blot images corresponding to Figure 9D.

Marker: Invitrogen, Cat # LC5615

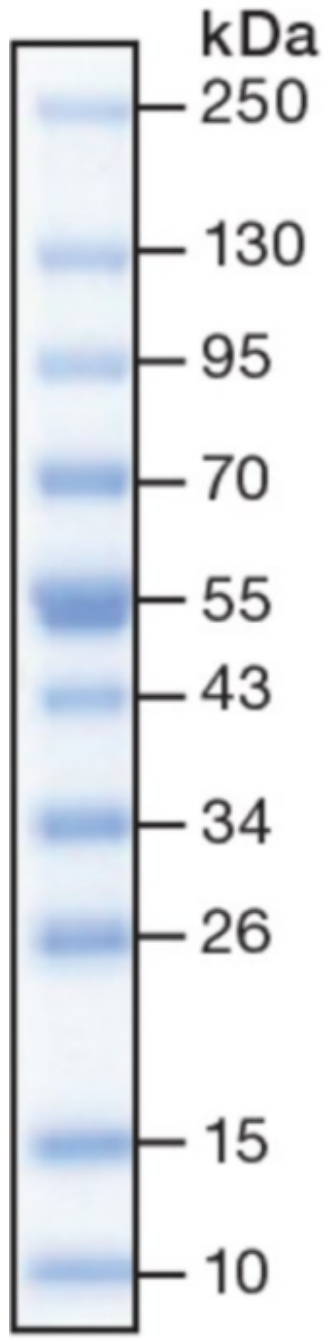

SLIRP

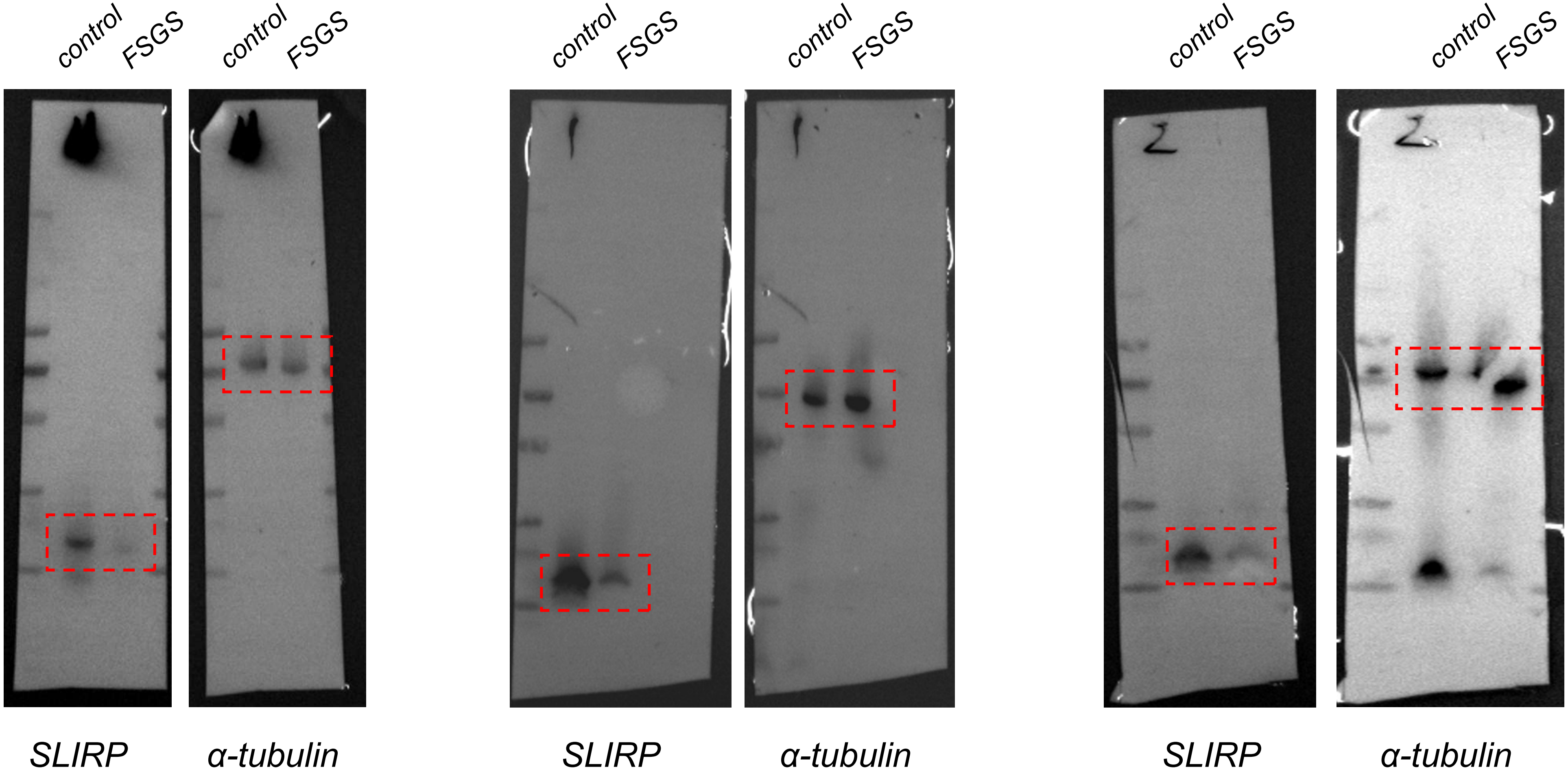
control
FSGS
control
FSGS
control
FSGS
control
FSGS
control
FSGS
control
FSGS
SLIRP
α-tubulin
SLIRP
α-tubulin
SLIRP
α-tubulin

BLVRB

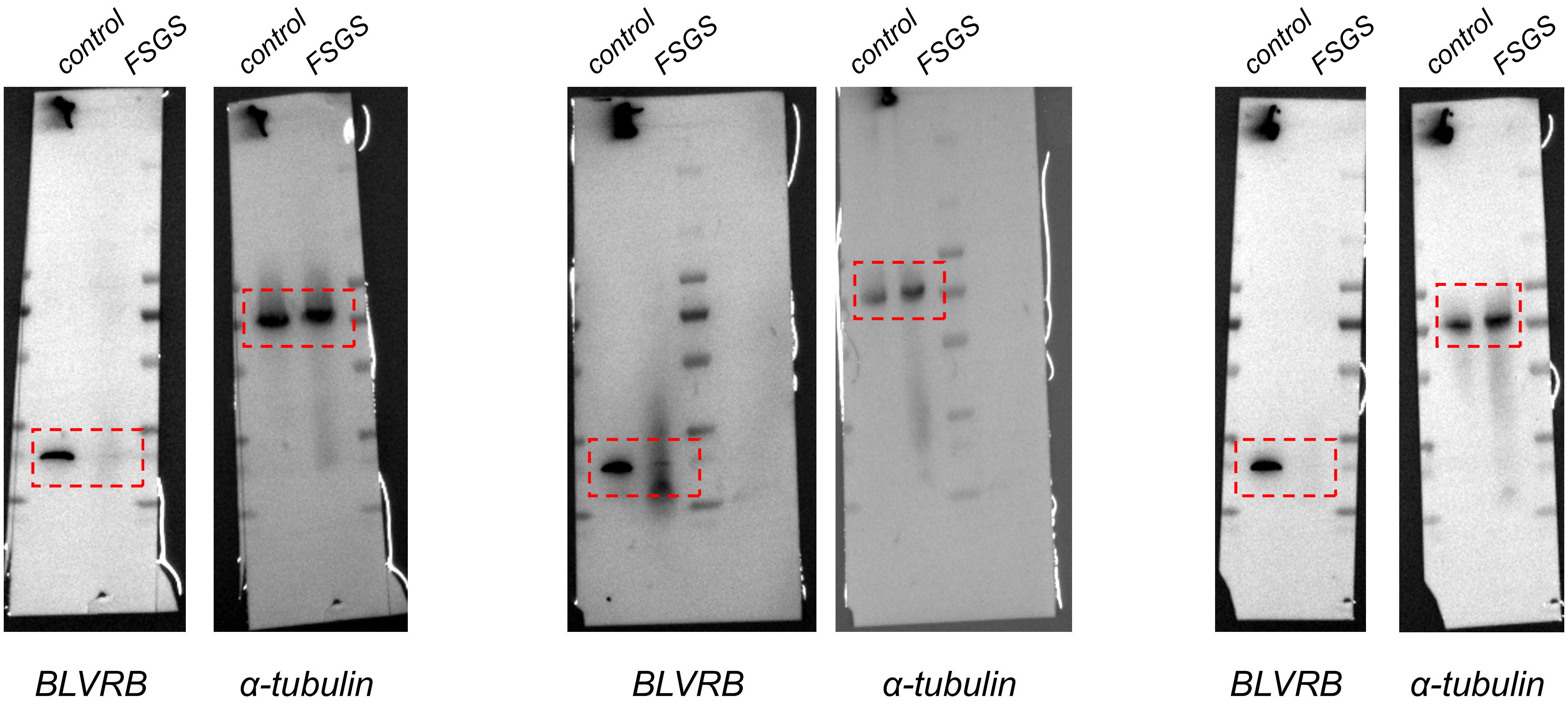
control
FSGS
control
FSGS
control
FSGS
control
FSGS
control
FSGS
control
FSGS
BLVRB
α-tubulin
BLVRB
α-tubulin
BLVRB
α-tubulin

ATP6V1F

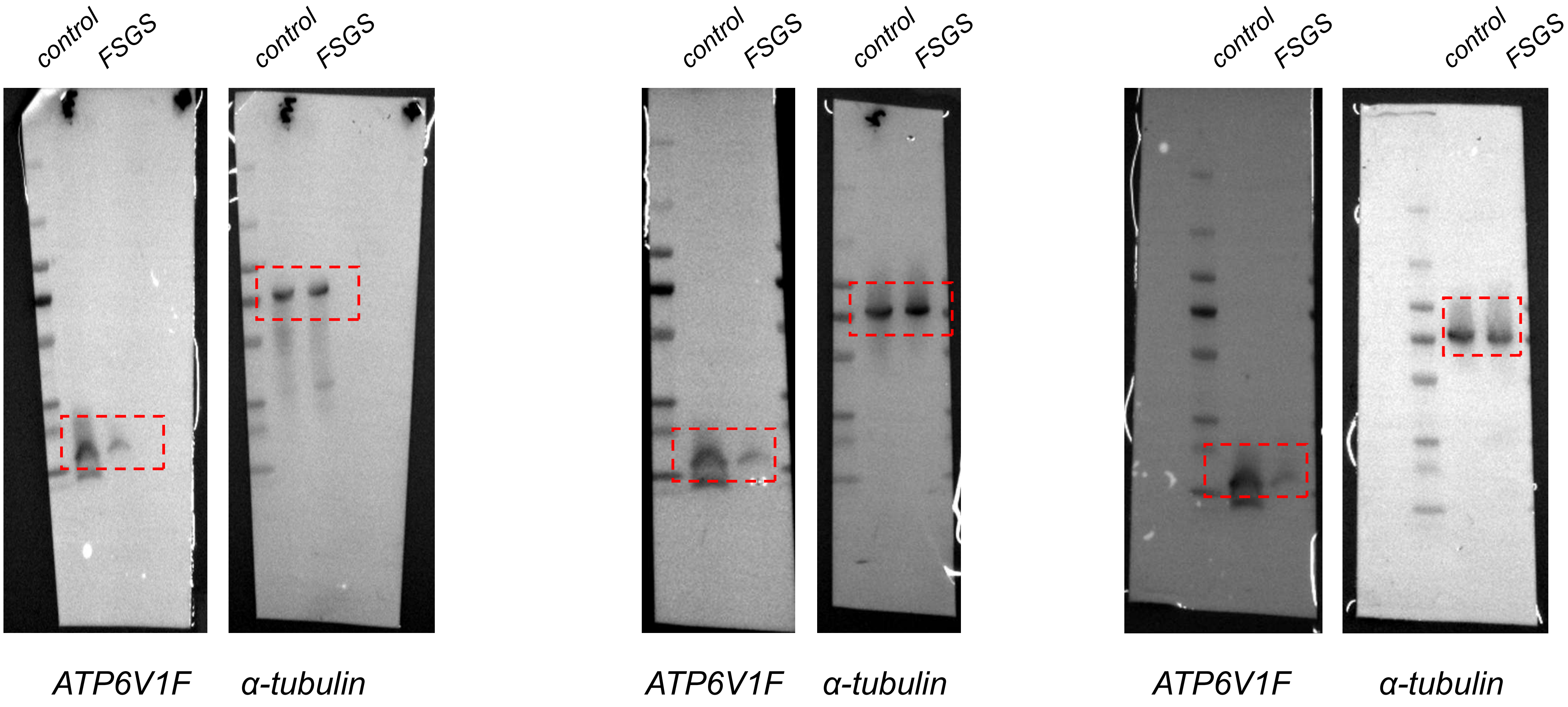
control
FSGS
control
FSGS
control
FSGS
control
FSGS
control
FSGS
control
FSGS
ATP6V1F
α-tubulin
ATP6V1F
α-tubulin
ATP6V1F
α-tubulin

COX5B

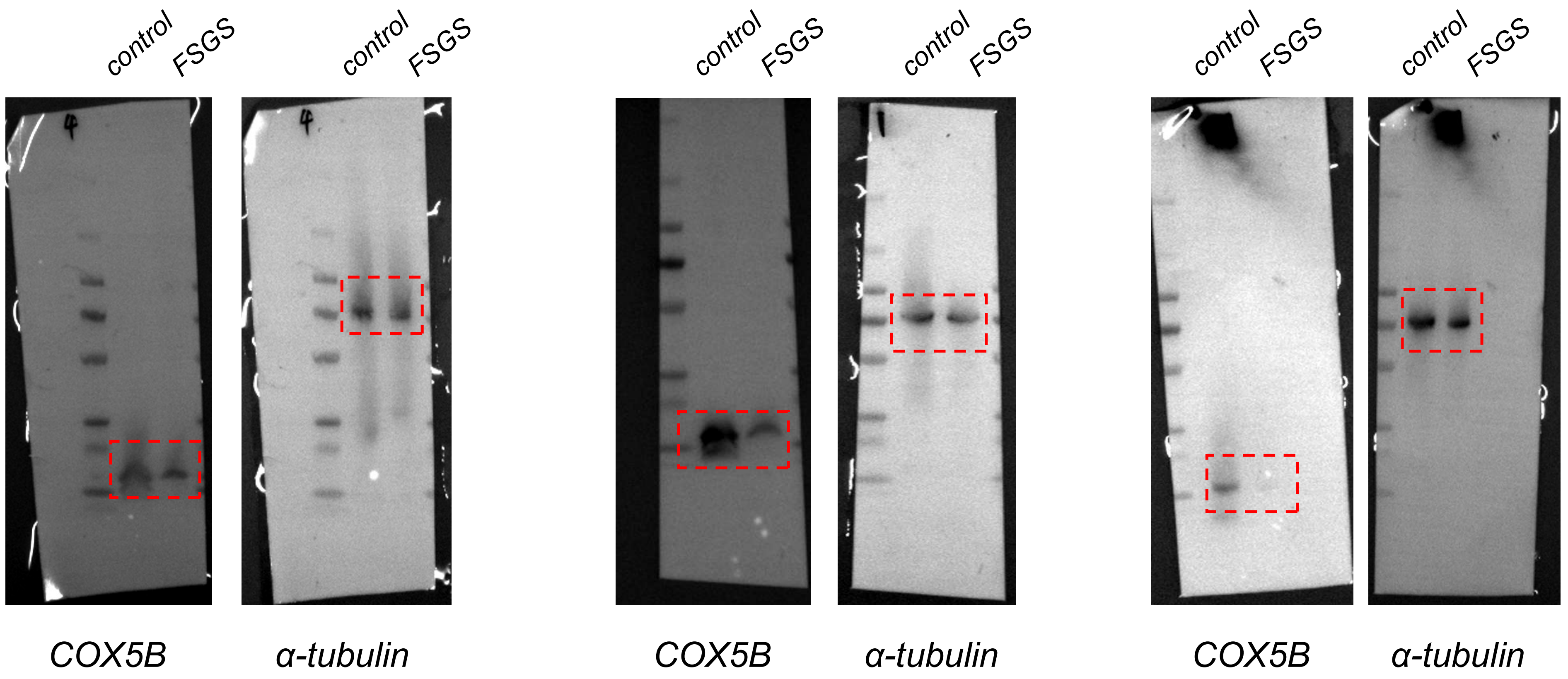
control
FSGS
control
FSGS
control
FSGS
control
FSGS
control
FSGS
control
FSGS
COX5B
α-tubulin
COX5B
α-tubulin
COX5B
α-tubulin

TMEM14A

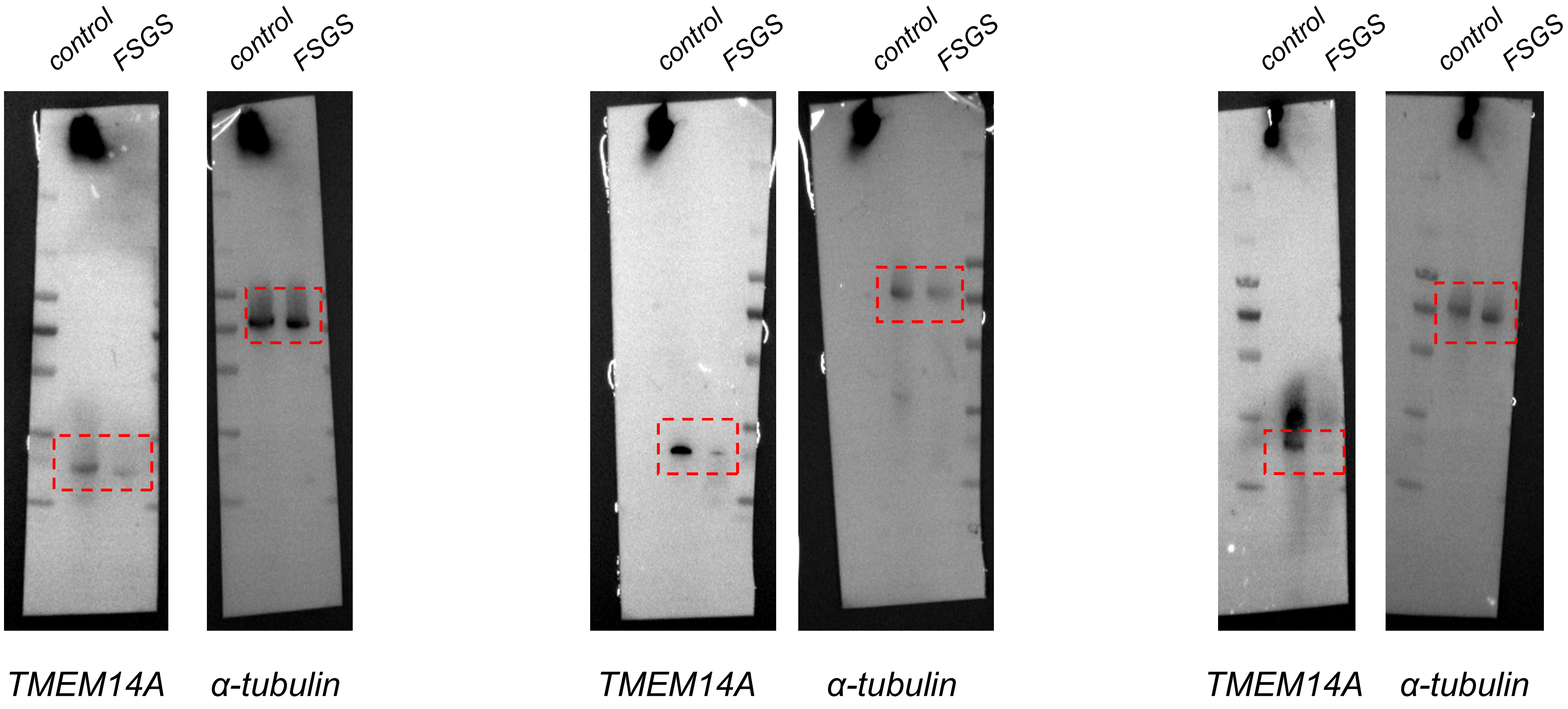
control
FSGS
control
FSGS
control
FSGS
control
FSGS
control
FSGS
control
FSGS
TMEM14A
α-tubulin
TMEM14A
α-tubulin
TMEM14A
α-tubulin

TRMT112

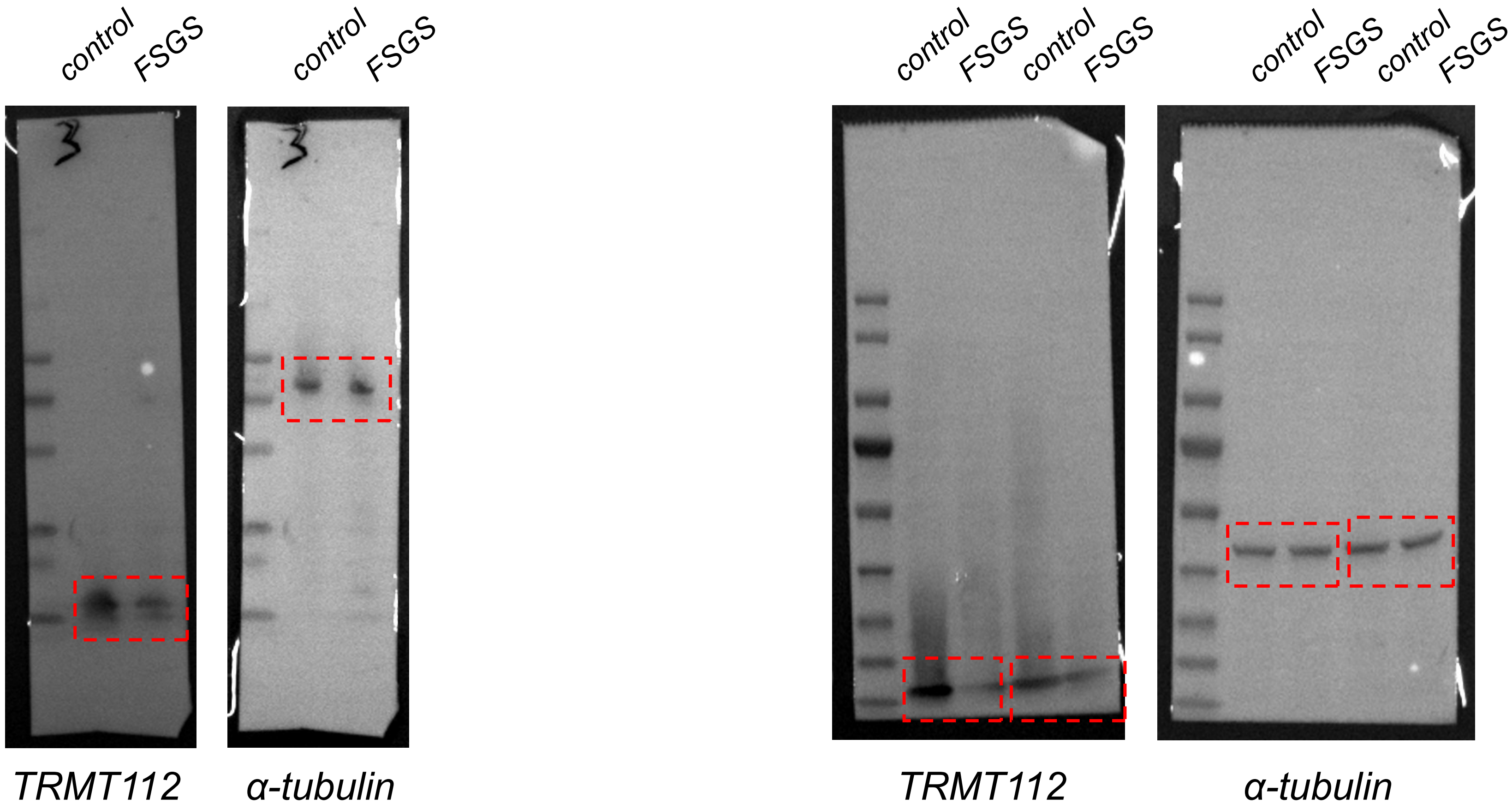
control
FSGS
control
FSGS
TRMT112
α-tubulin
control
FSGS
control
FSGS
control
FSGS
control
FSGS
TRMT112
α-tubulin
